# Modulation of synaptic plasticity, motor unit physiology, and TDP-43 pathology by CHCHD10

**DOI:** 10.1186/s40478-022-01386-9

**Published:** 2022-07-04

**Authors:** Tian Liu, Jung-A. A. Woo, Mohammed Zaheen Bukhari, Xinming Wang, Yan Yan, Sara Cazzaro Buosi, Aizara Ermekbaeva, Apoorva Sista, Peter Kotsiviras, Patrick LePochat, Ann Chacko, Xingyu Zhao, David E. Kang

**Affiliations:** 1 Department of Pathology, School of Medicine, Case Western Reserve University, Cleveland, OH 44106 USA; 2 Louis Strokes Cleveland VA Medical Center, Cleveland, OH USA; 3 Department of Molecular Medicine, Byrd Alzheimer’s Center & Research Institute, USF Health Morsani College of Medicine, Tampa, FL 33613 USA

**Keywords:** CHCHD10, TDP-43, Frontotemporal dementia, Mitochondria, Synapse

## Abstract

Mutations in *CHCHD10*, a gene coding for a mitochondrial intermembrane space protein, are associated with Frontotemporal dementia (FTD)-Amyotrophic lateral sclerosis (ALS) spectrum disorders, which are pathologically characterized by cytoplasmic inclusions containing TDP-43. FTD/ALS-linked CHCHD10 mutations and TDP-43 inclusions similarly induce mitochondrial defects in respiration, fusion/fission, mtDNA stability, and cristae structure, while sizeable amounts of cytoplasmic TDP-43 aggregates are found in mitochondria. However, the mechanistic link between CHCHD10 and TDP-43 pathogenesis remains unclear. In this study, we present immunohistochemical and biochemical evidence demonstrating that insoluble CHCHD10 aggregates accumulate and colocalize with phospho-TDP-43 inclusions in brains of FTLD-TDP and AD patients, and that insoluble CHCHD10 levels tightly correlate with insoluble TDP-43 levels in control and FTLD-TDP brains. In an experimental exploration of this pathological phenotype, transgenic mice neuronally expressing FTD/ALS-linked CHCHD10^R15L^ or CHCHD^S59L^ mutations but not CHCHD10^WT^ transgenic mice exhibit significantly increased CHCHD10 aggregation and phospho-TDP-43 pathology, which often colocalize within the same inclusions. Such pathologies are reflected in poor functional outcomes in long-term synaptic plasticity, motor unit physiology, and behavior in CHCHD10^R15L^ and CHCHD^S59L^ transgenic mice. In contrast, expression of CHCHD10^WT^ in hTDP-43 transgenic mice (TAR4;CHCHD10^WT^) significantly mitigates phospho-TDP-43 pathology and rescues TDP-43-induced impairments in synaptic integrity and long-term synaptic plasticity. In isolated mitochondria, the S59L mutation induces the aggregation of resident CHCHD10^S59L^ protein as well as the aggregation and slower turnover of recombinant TDP-43 imported into mitochondria. Likewise, in an in vitro cell-free system, the S59L mutation induces the aggregation of CHCHD10^S59L^ protein while simultaneously enhancing the aggregation of recombinant TDP-43, as evidenced by filter trap assays and atomic force microscopy. In contrast, recombinant CHCHD10^WT^ inhibits the growth of TDP-43 aggregates. These results in human brains, transgenic mice, and in vitro systems substantiate the role of wild type and mutant CHCHD10 in modulating mitochondrial CHCHD10 and TDP-43 pathogenesis together with associated phenotypes in long-term synaptic plasticity and motor unit physiology in mice and humans.

**Supplementary Information:**

The online version contains supplementary material available at 10.1186/s40478-022-01386-9.

## Introduction

The spectrum of Frontotemporal dementia (FTD) and Amyotrophic lateral sclerosis (ALS) disorders share multiple genetic and pathological etiologies. Specifically, proteopathic inclusions containing the TAR DNA binding protein-43 (TDP-43) represent the common pathological hallmark of FTD-ALS spectrum disorders [1–3]. TDP-43 inclusions are also common in Alzheimer’s disease (AD) brains, particularly in those with a more severe clinical phenotype [4, 5]. Pathological TDP-43 inclusions, which are typically phosphorylated (pSer409/410) [6, 7], aggregated, and mislocalized from the nucleus to the cytoplasm [8, 9], are invariably associated with severe mitochondrial abnormalities [10–14]. Sizeable amounts of cytoplasmic TDP-43 aggregates are colocalized with mitochondrial markers and found inside of mitochondria in human disease [15, 16]. Experimental studies have shown that the accumulation of TDP-43 in mitochondria of neurons induces mitochondrial dysfunction and synaptic damage [16–18], and inhibition of TDP-43 mitochondrial localization rescues mitochondrial abnormalities and neuronal death in cellular and animal models [16, 19, 20].

The gene *Coiled-coil-helix-coiled-coil-helix domain containing 10* (*CHCHD10*) encodes a small 15-kDa mitochondrial intermembrane space (IMS) protein, in which multiple mutations have been linked to the etiology of both sporadic and familial FTD-ALS spectrum disorders [21–24]. Previous studies have implicated the role of CHCHD10 in the regulation of mitochondrial respiration as well as maintenance of mitochondrial genome, cristae structure, and mitochondrial fusion [17, 21, 25, 26]. Specifically, CHCHD10 has been identified as a component of the mitochondrial contact site and cristae organizing system (MICOS) at least in part through its interaction with the core protein mitofilin, and patient-derived fibroblasts carrying the CHCHD10^S59L^ mutation exhibit disrupted MICOS complex and cristae structure [27, 28]. Soluble CHCHD10 levels decline in brains of FTLD-TDP patients and a mouse model of TDP-43 pathogenesis [26]. Interestingly, ectopic TDP-43 expression recapitulates the effects of FTD/ALS-linked CHCHD10 mutations in disrupting mitochondrial fusion, cristae integrity, and respiration, whereas CHCHD10^WT^ rescues these mitochondrial abnormalities induced by TDP-43 [26]. These changes in mitochondrial function, however, do not address whether and how CHCHD10 and TDP-43 proteinopathies per se are mechanistically related.

In this study, we found that insoluble CHCHD10 aggregates accumulate and colocalize with phospho-TDP-43 inclusions in brains of FTLD-TDP and AD patients, and insoluble CHCHD10 levels tightly correlate with insoluble TDP-43 levels in human brains. To experimentally explore this pathological finding, we assessed transgenic mice expressing wild type CHCHD10^WT^ or FTD/ALS-linked mutants (CHCHD10^R15L^ & CHCHD^S59L^) under the control of the neuron-specific *PrP* promoter. Herein, we present the first in vivo evidence that the FTD/ALS-linked CHCHD10^R15L^ and CHCHD10^S59L^ mutations drive CHCHD10 and TDP-43 proteinopathies originating from mitochondria, which directly mirror the functional changes in long-term synaptic plasticity and motor unit physiology. In contrast, CHCHD10^WT^ mitigates TDP-43 pathology and rescues TDP-43-induced impairment in long-term synaptic plasticity. In vivo alterations in CHCHD10 and TDP-43 proteinopathies by CHCHD10 variants are recapitulated in primary neurons, cultured cells, isolated mitochondria, and purified recombinant proteins, which collectively indicate that CHCHD10 variants directly regulate the solubility and aggregation of CHCHD10 and TDP-43 in mitochondria.

## Materials and methods

### Ethics approval

All the experiments methods and protocols involving mice in this study were approved by Institutional Animal Care and Use Committee (IACUC), and all methods were performed in accordance with the relevant guidelines and regulations have also been approved by IACUC and Institutional Biosafety Committees (IBC).

### Human brain samples

Frozen tissues and floating brain sections from the frontal gyrus of FTLD-TDP, AD, and nondemented controls were obtained from Dr. Allan Levey and Dr. Marla Gearing at Emory Alzheimer’s Disease Research Center (ADRC). Pathology-confirmed FTLD-tau, AD, and nondemented control tissues were matched as closely as possible for sex, age, and APOE genotypes during the procurement phase by the Emory ADRC (P50 AG025688) (Additional file 1: Fig. S1).

### Mice

Wild type C57BL6, CHCHD10^WT^, CHCHD10^R15L^, and CHCHD10^S59L^ mice [26] as well as TAR4 mice expressing human wild type TDP-43 [29] were bred in the C57BL6 background. CHCHD10 variant transgenic mice and CHCHD10 expression profiles have previously been described [26]. Mice were housed together in a SPF facility with 2–4 littermates in sterile plastic cages with pelleted bedding until euthanization by isoflurane overdose and cervical dislocation or transcardial perfusion. Water and food were supplied ad libitum with 12-h light/dark cycle and provided enrichment with a mouse igloo. Available mouse littermates were allocated to experimental groups based on age and genotypes for specific experiments. Individuals directly conducting studies on mice were blinded to their genotypes. All mice exhibited normal health without overt abnormalities until the time of euthanization. Detailed methods of behavioral testing and electrophysiological (ex vivo & in vivo) studies are described in Supplemental Methods.

### Antibodies and reagents

Mouse Anti-Flag M2 (Cat#: F3165) and β-actin (Cat#: A2228) monoclonal antibodies were obtained from Sigma-Aldrich (St. Louis, MO, USA). Anti-TDP-43 (G400, Cat#: 3448) antibody was purchased from Cell Signaling (Danvers, MA, USA). Anti-human TDP-43 antibody (Cat#: H00023435-M01) was purchased from Abnova (Walnut, CA, USA). Anti-Phospho TDP-43 (pS409/410) (Cat#: TIP-PTD-M01) was ordered from Cosmo Bio (Tokyo, Japan). Anti-CHCHD10 (Cat#: ab121196), PSD95 (Cat#:ab18258) and synaptophysin (ab14692) antibodies were obtained from Abcam (Cambridge, UK). Anti-Tom20 (Cat#: sc-17764), Lamin B1(Cat#:sc-56144) and mitofilin (Cat#: sc-390706) antibodies were purchased from Santa Cruz Biotechnology (Dallas, TX, USA). Anti-GLUR2 (Cat#: ABIN3031067) antibody was purchased from Antibodies-online (Limerick, PA, USA). Mouse anti-NeuN (MAB377) antibody was purchased from Millipore Sigma (Burlington, MA, USA).

### Atomic force microscopy

CHCHD10^WT^ and CHCHD10^S59L^ recombinant proteins (1 μM) were incubated by themselves or with recombinant full-length human TDP-43 (1 μM) at 37 °C for 8 h with vigorous shaking (1200 rpm), after which samples were subjected to atomic force microscopy (AFM) and filter trap assay (FTA). For AFM imaging, 10 μλ protein solution was dropped on freshly cleaved mica surface. After 20 min adsorption, filtered distilled water was used to gently wash away excessive solution. The mica was incubated at room temperature until dry followed by AFM imaging. The AC-mode of AFM imaging was used on a JPK NanoWizard 4 AFM (Bruker, USA) under ambient conditions. Commercial PPP-NCHAuD cantilevers (Nanosensors, Switzerland) with nominal spring constant of 42 N/m and resonant frequency of 330 kHz were used. Particle size was estimated by measuring the area of high-contrast particles > 10 nm in height using Image J (NIH, Bethesda, MD, USA).

### Recombinant proteins

Recombinant human TDP-43 was purchased from R&D Systems (Minneapolis, MN, USA). CHCHD10^WT^ and CHCHD10^S59L^ were subcloned into pET22B vector containing the HIS tag, transformed into Rosetta competent cells and grown in a 37 °C shaker at 250 rpm until OD^600^ = 0.6–0.9, induce with 1 mM IPTG at 20 °C (250 rpm) for 24 h. Rosetta cells were collected by centrifugation at 4 °C and lysed in lysis buffer (Tris 20 mM, pH8.0, NaCl 150 mM, imidazole 10 mM, Gendepot 1X protease inhibitor cocktail) followed by sonication on ice. Supernatant was collected after centrifugation and shaken for 1 h at 4 °C with GE Healthcare Ni Sepharose. Bound proteins on sepharose were washed 3 times with ice-cold lysis buffer, and recombinant proteins were eluted with ice-cold elution buffer (Tris 20 mM, NaCl 150 mM, 200 mM imidazole), after which proteins were dialyzed in dialysis buffer (Tris 20 mM, NaCl 150 mM, DTT 1 mM) at 4 °C overnight.

### Cell culture

HEK293T and Tet-inducible TDP-43 Hela cells [1] were cultured in Dulbecco’s modified Eagle’s medium (DMEM, Thermo Scientific, MA, USA) supplemented with 10% fetal bovine serum (FBS) and 1% penicillin/streptomycin (P/S). Mouse cortical primary neurons from P0 pups were cultured in Neurobasal medium with 1 × B-27 supplement and 1 × L-Glutamine (Invitrogen, CA, USA) in humidified atmosphere (5% CO_2_) at 37℃ as previously described [2, 3].

### Rotarod and grip strength

Mice were individually housed one week prior to behavioral testing. Each mouse was handled for at least 2 min in that week. On the day of behavioral testing, the mice were moved to behavior room and allowed to acclimate for at least 30 min before trials began. For the rotarod test, rotarod machine (Panlab, rotarod Model LE8205) was set with speeds of 4–40 rpm and 600 s acceleration time. Mice were brought into the room in groups of 3–5 for testing. Mice were given two minutes of practice trials at an accelerating speed starting from 4 rpm to learn the behavior. During the four trials with accelerating mode (4–40 rpm in 5 min max), speeds and times to fall were recorded. For the grip strength, the test was performed with the Ugo Basile grip strength meter (Cat.47200). Mice were put on the mesh platform on all 4 legs, and the tail was pulled slowly and gently until the mouse lost grip of the mesh, and peak gram force was recorded.

### Ex vivo slice recordings

Brain slices from 10-month-old WT, CHCHD10^WT^, CHCHD10^R15L^, CHCHD10^S59L^, TAR4 and TAR4; D10^WT^ mice were subjected to input–output (I-O) curves, paired-pulse facilitation (PPF), and long-term potentiation (LTP) as previously described [4]. The stimulating electrode was placed in the Schaffer collaterals of the hippocampus with the ACSF flow rate of 1 ml/min at 30 ± 0.5 °C. The recording glass electrode was positioned at the CA1 stratum radiatum below the pyramidal cell layer and the stimulating pulses were generated by the Digidata 1440 A interface (Molecular Devices, Sunnyvale, CA, USA) and a stimulus isolator (model 2200; A-M Systems, Sequim, WA, USA) controlled by Clampex 10.6 software (Molecular Devices). Field excitatory postsynaptic potentials (fEPSPs) were amplified using a differential amplifier (model 1800; A-M Systems), filtered at 1 kHz, and digitized at 10 kHz. I-O curves were generated by stepping stimulation amplitude that elicited half-maximal fEPSP from 1 to 15 mV at the rate of 0.05 Hz. PPF was evoked by two pulses with interpulse intervals from 20 to 300 ms. Percentage of the facilitation was calculated by dividing fEPSP slope elicited by the second pulse with the fEPSP slope elicited by the first pulse. LTP was induced by theta burst stimulation (five trains of four pulses at 200 Hz separated by 200 ms, repeated six times with an inter-train interval of 10 s) and sampled for 60 min after the induction. LTP was calculated by dividing the slope of 60 min post-induction responses with the average slope of 20 min baseline responses.

### In vivo sciatic nerve motor unit recordings

Mice were anesthetized with inhaled isoflurane and placed in the prone position on a thermostatic warming plate. The ring of recording electrode was placed on the skin overlying the proximal portion of the gastrocnemius muscle of the hind limb. The ring of reference electrode was placed on the skin over mid-metatarsal portion of the foot. The distance between these two ring electrodes was 10 mm. Electrode gel was applied to the skin underlying the ring electrodes to reduce impedance and maximize ring electrode–skin contact. The stimulating electrodes were two 27G monopolar needle electrodes. The cathode monopolar electrode was placed close to the sciatic never at the proximal thigh, and the anode was inserted about 10 mm proximally in the subcutaneous tissue overlying the sacrum. A monopolar needle electrode was placed on the contralateral hind limb or tail as the ground electrode. The distance between the stimulating and recording electrodes was 12–16 mm. A square-wave pulse with 0.1 ms duration and1–10 mA intensity was delivered to induce compound muscle axon potential (CMAP). Stimulus intensity was increased until the CMAP was maximized, which represents the total output from a muscle group. The constant-current stimulus of 0.1 ms duration was sent to evoke a motor response in 0.025 mA incremental steps at 0.1 Hz until a small all-or-none response was evoked. Each incremental response is stable and should be visually distinct and larger compared with the preceding response. The stimulus intensity was increased until the CMAP increased in a quantal fashion. This process was repeated for a total of 10 increments. Individual motor unit amplitudes were obtained by subtracting the amplitude of each response from that of the prior response. The individual values were averaged to yield the single motor unit potential (SMUP). Maximum CMAP was divided by SMUP to yield motor unit number estimation (MUNE).

### Isolation of mitochondria, mitochondrial fractionation, and mitochondrial TDP-43 turnover

Mitochondria Isolation Kit for cultured cells as well as Mitochondria Isolation Kit for Tissue (Thermo Scientific, MA, USA) were used for Mitochondria isolation following the manufacturer’s instructions. For mitochondrial import assays, HEK293T cells were transfected with Flag-CHCHD10^WT^, Flag-CHCHD10^R15L^, or Flag-CHCHD10^S59L^ for 48 h. Intact mitochondria were isolated, aliquoted and resuspended in 100 μλ of mitochondria import buffer (250 mM sucrose, 10 mM MOPS-KOH, pH 7.2, 80 mM KCl, 5 mM MgCl_2_, 2 mM ATP, 2 mM NADH, 3% BSA) on ice. Then 2 μγ recombinant human TDP-43 (R&D System, Minneapolis, MN, USA) was added to each tube at 33 °C for 1 h. Then the mitochondria were spun down at 12000 g for 10 min at 4 °C, resuspended in 100 μλ new import buffer after washing with PBS. Mitochondria were then placed back at 33 °C for 0, 1, 4, 8, and 12 h, after which mitochondria were collected by centrifugation and lysed with RIPA to separate the RIPA-soluble and RIPA-insoluble fractions. After which fractions were subjected to Western blotting and/or filter-trap assay.

Fractionation of isolated mitochondria was performed using a previously documented method [5] with minor modifications. Briefly, isolated mitochondria were washed with swelling buffer (10 mM KH2PO4, pH 7.4) followed by resuspension in swelling buffer for 20 min at 4 °C. Then the equal volume of shrinking buffer (10 mM HEPES, pH 7.4, 32% sucrose, 30% glycerol, 10 mM MgCl2) was added and resuspended for 15 min followed by centrifugation at 10,000 g for 15 min at 4 °C. Both pellet (mitoplast) including inner membrane (IM) and matrix, and supernatant including outer membrane (OM) and intermembrane space (IMS) fractions were obtained. The supernatant was centrifuged at 100,000 *g* for 40 min at 4 °C to separate OM and IMS fractions. The pellet (mitoplast) was washed twice with washing buffer (250 mM sucrose, 1 mM EGTA, 10 mM HEPES, pH 7.4), resuspended in swelling buffer, sonicated and subjected in centrifugation at 12,000 *g* for 15 min. The supernatant was centrifuged at 100,000*g* for 40 min at 4 °C to separate IM and matrix fractions.

### DNA constructs, transfections, and rAAV9 transduction

P3X-Flag-CHCHD10^WT^, P3X-Flag-CHCHD10^R15L^, and P3X-Flag-CHCHD10^S59L^ constructs have previously been described [17]. The generation and production of rAAV9 virus of TDP-43-tomato-HA has previously been described [17]. For DNA plasmid transfections, HEK293T and Hela cells were transfected with fugene HD (Promega, Madison, WI, USA) in Opti-MEM I (Invitrogen, Carlsbad, CA, USA) according to the manufacturer’s instructions and harvested 48 h post-transfection. Primary neurons were transduced with rAAV9 virus of TDP-43-tomato-HA on DIV7 and fixed on DIV21 for immunocytochemistry.

### Cell/tissue lysis, SDS-PAGE, and filter-trap assaym

Cells and brain tissues were lysed using RIPA lysis buffer (50 mM Tris pH 7.4, 150 mM NaCl, 2 mM ethlenediaminetetra acetic acid, 1% NP-40, 0.1% sodium dodecyl sulfate). Total protein concentrations were quantified by a colorimetric detection assay (BCA Protein Assay, Pierce, USA). Equal amounts of protein lysates were subjected to sodium dodecyl sulfate–polyacrylamide gel electrophoresis and transferred to nitrocellulose membrane (Millipore Corporation, Bedford, MA, USA). For filter-trap assays (FTA), equal amounts of RIPA-soluble or sonicated RIPA-insoluble extracts o were filtered through 0.2 μm of cellulose acetate membranes (ThermoFisher Scientific, MA, USA) placed in a 96-well vacuum dot blot apparatus (Bio-Rad, Hercules, CA, USA) [30] followed by PBS washing and 20% methanol fixation. Interested proteins were probed by primary antibodies after blocking with 5% skim milk and corresponding peroxidase-conjugated secondary antibodies, followed by detection by ECL (Merck Millipore Corporation, Darmstadt, Germany). All immunoblot images were acquired by LAS-4000 (GE Healthcare Biosciences, Pittsburgh, PA) and quantified using ImageJ (NIH, Bethesda, MD).

### Immunocytochemistry, immunohistochemistry, and fluorescent microscopy

For immunocytochemistry (ICC), primary neurons were washed with PBS and fixed with 4% paraformaldehyde (PFA) for 15 min at room temperature. Fixed cells were washed with PBS and incubated with blocking solution (0.2% Triton X-100, 3% normal goat serum) for 1 h, incubated with primary antibody at 4 °C overnight, washed with PBST 3X, and incubated with Alexa-488 or Alexa-594 conjugated secondary IgG antibodies for 1 h at room temperature (Vector Laboratories, Burlingame, CA). Slides were then washed three times with PBST and mounted with fluorochrome mounting solution (Vector Laboratories). For immunohistochemistry (IHC), mice were perfused with PBS, and half brains/spinal cords were immediately stored at − 80℃ for biochemical analysis, and the other half brain and lumbar spinal cord were fixed with 4% paraformaldehyde at 4 ℃ for 24 h followed by cryoprotection in 30% sucrose. Prior to staining for CHCHD10, Flag-CHCHD10 (M2), mitofilin, TDP-43, or pTDP-43, antigen retrieval (10 mM sodium citrate, 0.05% Tween 20, Ph 6.0) was performed for 5 min at 95 ℃. Thirty-micron sections were blocked using normal goat serum for 1 h and subjected to primary antibodies at 4 ℃ overnight, followed by 1 h incubation with secondary antibody (Alexa-594 and Alexa-488) at room temperature prior to mounting as previously described [31]. For IHC experiments using human brain tissue, post-treatment with TrueBlack (Biotium, Fremont, CA) was performed prior to mounting. Briefly, tissues were incubated with 1X TrueBlack (diluted with 70%) ethanol for 1 min and washed with PBS three times. And then the tissues were mounted to the slides for imaging. Olympus FV10i confocal microscope (Tokyo, Japan) or ZEISS LSM880 confocal microscope was used to capture all the images, and the ImageJ software (NIH, Bethesda, MD) was used to quantify the immunoreactivities. In ICC and IHC experiments, all comparison images were acquired with identical laser intensity, exposure time, and filter. Regions of interest were chosen randomly, and investigators were blinded to genotypes of mice and experimental conditions during image acquisition and quantification. Adjustments to the brightness/contrast were applied equally to all comparison images. Intensities for synaptophysin from hippocampal CA3 and TDP-43 and pS409/410-TDP-43 from the frontal cortex were quantified using ImageJ (NIH, Bethesda, MD). Colocalizations were measured with Image J via the JACoP plugin for Manders overlap coefficient (MOC). The same thresholds for each channel were used when analyzing the coefficient.

### Statistical analysis

All graphs were analyzed and made using GraphPad Prism 6.0 software (GraphPad Software, San Diego, CA, USA) using student’s *t*-test, 1-way analysis of variance (ANOVA) followed by Tukey post hoc test, 2-way ANOVA followed by Tukey post hoc test, or linear regression and Pearson’s correlation as described in figure legends. Differences were deemed significant when *P* < 0.05. All graphs were expressed as mean ± S.E.M (error bars).

## Results

### Insoluble CHCHD10 aggregates accumulate and colocalize with phospho-TDP-43 inclusions in brains of FTLD-TDP and AD patients

**Fig. 1:**
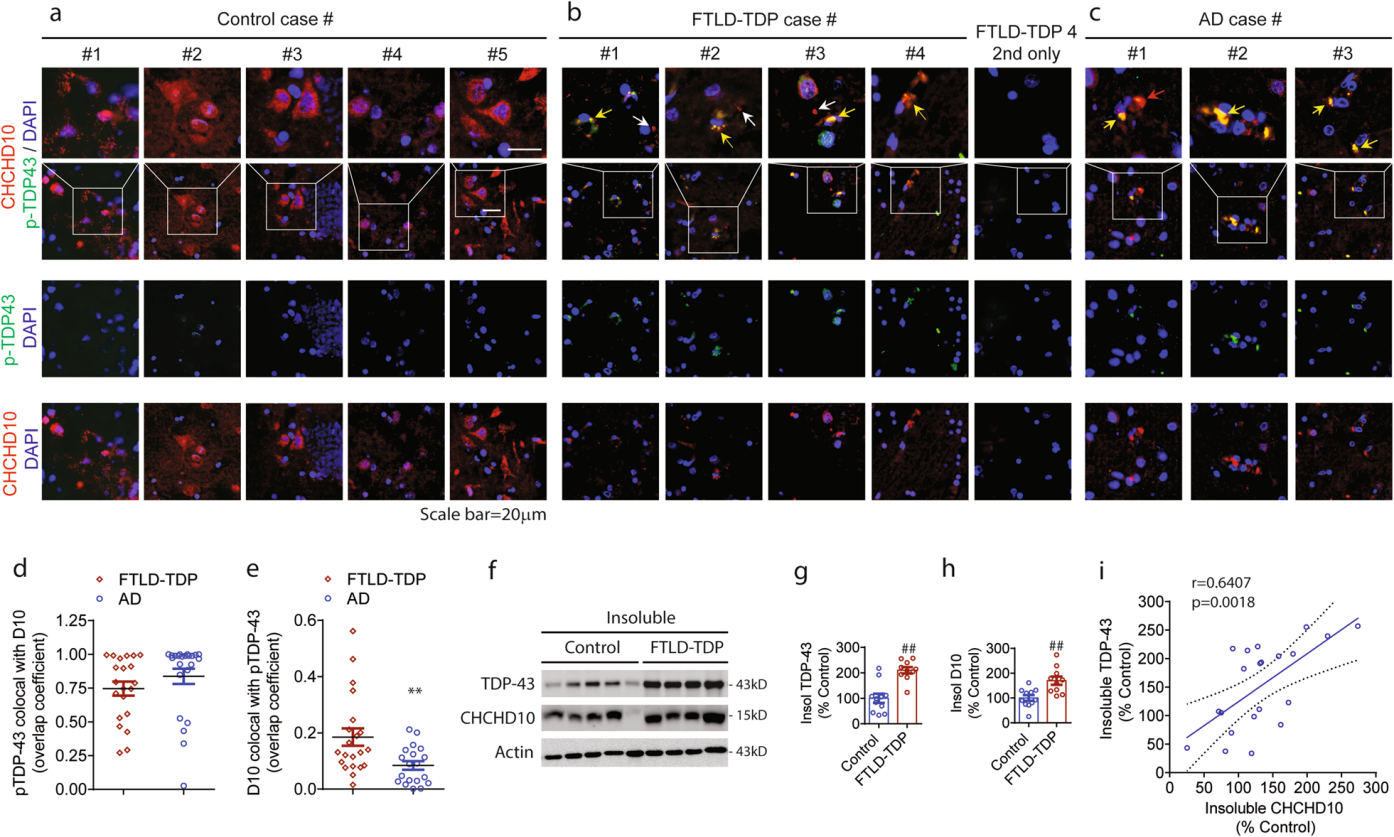
Insoluble CHCHD10 aggregates accumulate and colocalize with phospho-TDP-43 inclusions in FTLD-TDP and AD patients’ brains. **a**–**c** Representative images of postmortem frontal cortex brain sections from healthy controls, FTLD-TDP, and AD cases immunostained for pS409/410-TDP-43 (green), CHCHD10 (red), and DAPI (blue). White boxes magnified in bottom panels. Colocalization of CHCHD10 with phospho-TDP-43 (yellow arrows), no colocalization (white arrows), and CHCHD10 aggregates larger than phospho-TDP-43 (red arrow). **d**, **e** Quantification of the pS409/410-TDP-43 (green) colocalization with CHCHD10 (red) (d), and CHCHD10 (red) colocalization with pS409/410-TDP-43 (green) (e) using Mander’s overlap coefficient (MOC, ImageJ) from FTLD-TDP and AD frontal cortex (t-test, **p < 0.01, n = 22 images from 4 FTLD-TDP cases, n = 19 from 3 AD cases). **f** Representative blots of TDP-43, CHCHD10, and actin from RIPA-insoluble frontal cortex brain extracts. **g**, **h** Quantification of RIPA-insoluble TDP-43 (l) and insoluble CHCHD10 (m) from human FTLD-TDP and nondemented control frontal cortex (t-test, ##P < 0.0001, n = 10 FTLD-TDP, n = 11 control). **i** Positive correlation between insoluble TDP-43 and insoluble CHCHD10 from FTLD-TDP and control frontal cortex (Linear regression, F = (1, 19) = 13.23, R^2^ = 0.4104, P = 0.0018; Pearson’s r = 0.6407, n = 21). Blue line represents the slope and dashed lines the 95% confidence interval

We recently reported that detergent soluble CHCHD10 significantly declines in brains of FTLD-TDP patients and TDP-43 transgenic mice [26]. To assess this finding in a different way, we performed immunohistochemical staining for CHCHD10 together with phospho-TDP-43 in the frontal cortex of 4 frontotemporal lobar degeneration-TDP (FTLD-TDP) cases, 5 nondemented control cases, and 3 Alzheimer’s disease (AD) cases using TrueBlack® to eliminate background autofluorescence (Case information: Additional file 1: Fig. S1a). As expected, nondemented controls showed little to no phospho-TDP-43 but salient CHCHD10 staining, the latter which was distributed throughout the neuronal cytoplasm and neuropil (Fig. 1a). In contrast, FTLD-TDP cases exhibited marked cytoplasmic phospho-TDP-43 staining, which was absent in FTLD brains stained only with secondary antibodies (Fig. 1b). Surprisingly, the pattern of CHCHD10 staining in FTLD-TDP brains differed markedly from that of controls, as the broad distribution of cytoplasmic CHCHD10 staining was diminished; instead, CHCHD10 immunoreactivity showed an asymmetric aggregate-like pattern that often colocalized with phospho-TDP-43 inclusions (Fig. 1a). Nearly all larger CHCHD10 aggregates colocalized with phospho-TDP-43 (Fig. 1b, yellow arrows), whereas smaller CHCHD10 puncta often did not contain phospho-TDP-43 (Fig. 1b, white arrows). Like that seen in FTLD-TDP, AD brains also demonstrated CHCHD10 aggregate-like staining, most of which colocalized with phospho-TDP-43 cytoplasmic inclusions (Fig. 1c, yellow arrows). Interestingly, some CHCHD10 aggregates that colocalized with TDP-43 were larger than phospho-TDP-43 inclusions (Fig. 1c, red arrow). Overall, Manders overlap coefficient (MOC) for phospho-TDP-43 colocalization with CHCHD10 reached 0.75 in FTLD-TDP and 0.84 in AD with no significant difference between them (Fig. 1d), indicating that most phospho-TDP-43 inclusions contain CHCHD10 in both FTLD-TDP and AD. Conversely, MOC for the proportion of CHCHD10 colocalizing with phospho-TDP-43 was 0.18 in FTLD-TDP and 0.08 in AD (Fig. 1e), indicating that while only ~ 18% of CHCHD10 colocalizes with phospho-TDP-43 in FTLD-TDP, a significantly larger proportion of CHCHD10 colocalizes with phospho-TDP-43 in FTLD-TDP versus in AD.

To determine if the observed aggregate-like staining in FTLD-TDP is detergent insoluble, we extracted the RIPA-insoluble fraction from the frontal cortex of control and FTLD-TDP brains. RIPA-insoluble fraction of FTLD-TDP brains indeed exhibited significant 2.1-, 1.7-, and 2.4- fold increases in insoluble TDP-43, CHCHD10, and phospho-TDP-43, respectively, compared to those in controls (Fig. 1f-h, Additional file 1: Fig. S1b,c), confirming the insolubility of both aggregates. Pearson’s correlation analysis demonstrated a significant positive correlation between insoluble TDP-43 and insoluble CHCHD10 in control and FTLD-TDP brains with Pearson’s *r* = 0.6407 (Fig. 1i), indicating a pathological correlation.

### FTD/ALS-linked CHCHD10 mutants drive CHCHD10 and TDP-43 pathogenesis in the CNS

Based on the preceding observations in post-mortem human brains, we next tested the hypothesis that FTD/ALS-linked CHCHD10 mutants, which themselves are prone to aggregation [32, 33], can drive both CHCHD10 and TDP-43 proteinopathies. For this purpose, we utilized wild type (WT) and CHCHD10 transgenic mouse variants, in which N-terminally Flag-tagged wild type (CHCHD10^WT^) or FTD/ALS-linked mutants (CHCHD10^R15L^ or CHCHD10^S59L^) are expressed in the central nervous system (CNS) under control of the neuron-specific mouse *PrP* promoter [26]. And majority of cells overexpressing CHCHD10^WT^, CHCHD10^R15L^ or CHCHD10^S59L^ in these mice brain are neurons (Additional file 2: Fig. S2c). These mice (D10-WT, R15L, or S59L) express CHCHD10 transcripts at 150–180% above the endogenous mouse CHCHD10 transcript, and human CHCHD10 transgenes are expressed throughout all regions of the brain and spinal cord [26]. We have previously shown that Flag-CHCHD10 localizes nearly exclusively to mitochondria like endogenous CHCHD10 [17]. As endogenous CHCHD10 is known to be targeted primarily to the mitochondrial intermembrane space (IMS) via the Mia40 pathway [34], we tested the sub-mitochondrial localization of Flag-CHCHD10 by performing mitochondrial isolation followed by sub-mitochondrial fractionation to separate the outer membrane (OM), intermembrane space (IMS), inner membrane (IM), and matrix from 3 Flag-CHCHD10^WT^ transgenic mice and a littermate WT mouse. Indeed, the vast majority of Flag-CHCHD10 (~ 72%) localized to the intermembrane space (IMS) as expected, demonstrating correct targeting and sub-mitochondrial localization of Flag-CHCHD10 (Additional file 2: Fig. S2a,b).

**Fig. 2:**
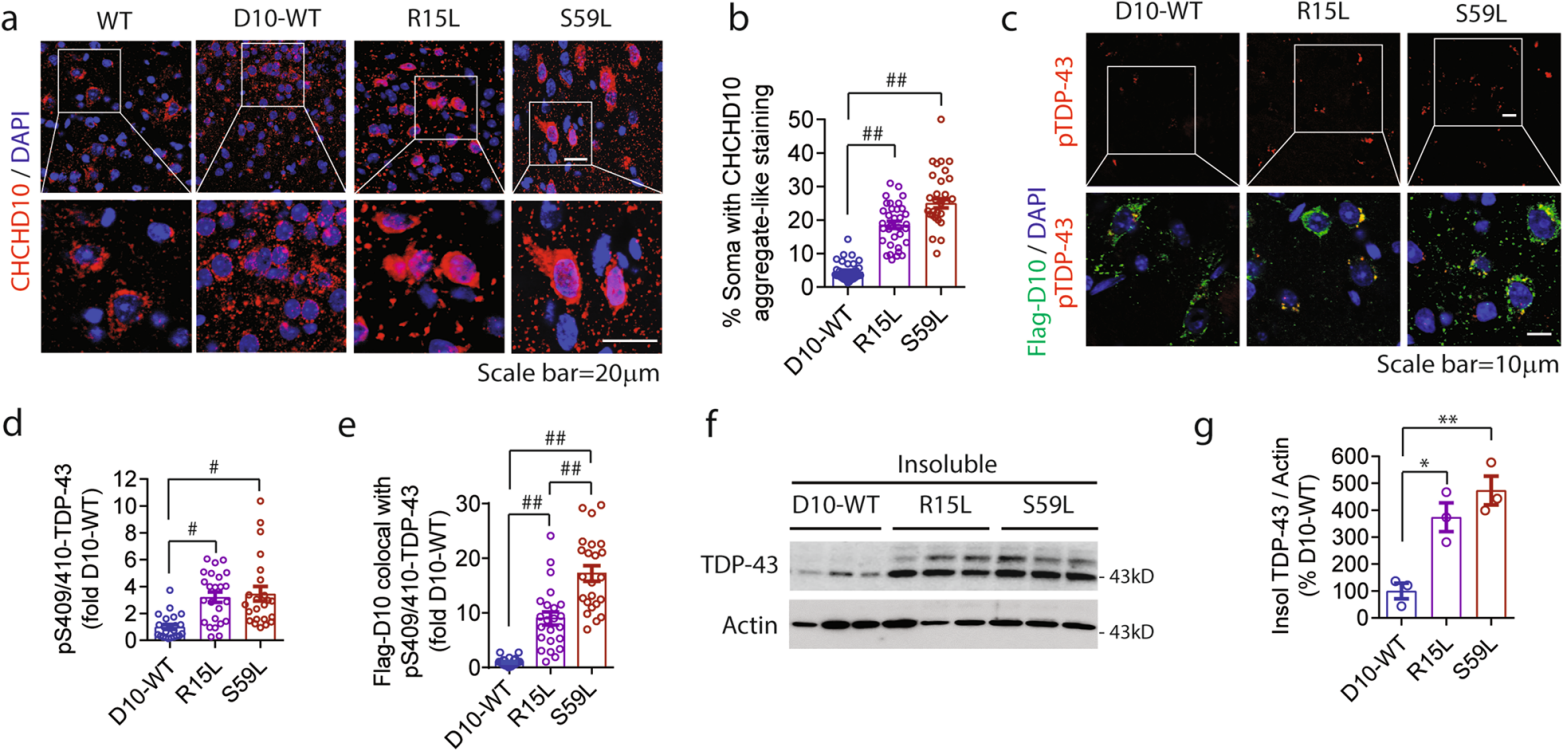
FTD/ALS-linked CHCHD10 mutants drive both CHCHD10 and TDP-43 pathogenesis in the CNS. **a** Representative images of brain sections from 10-month-old WT, CHCHD10^WT^, CHCHD10^R15L^, and CHCHD10^S59L^ mice immunostained for CHCHD10 in the frontal cortex. White boxes magnified in bottom panels. **b** Quantification of the percentage of soma in frontal cortex from Fig. (2a) with aggregated CHCHD10 (1-way ANOVA, F(2, 104) = 101.9, P < 0.0001: posthoc Tukey, ##p < 0.0001, n = 34–37 images/genotype from 4 mice/genotype). **c** Representative images of brain sections from 10-month-old CHCHD10^WT^, CHCHD10^R15L^, and CHCHD10^S59L^ mice immunostained for pS409/410-TDP-43 (green) and DAPI (blue). White boxes magnified in bottom panels. **d** Quantification of pS409/410-TDP-43 inclusions from Fig. (2c) (1-way ANOVA, F(2, 67) = 11.52, P < 0.0001: posthoc Tukey, #p < 0.005, n = 23–24 images/genotype from 4 mice/genotype). **e** Quantification of Flag-CHCHD10 colocalization with pS409/410-TDP-43 from Fig. (2c) (1-way ANOVA, F(2, 66) = 56.56, P < 0.0001: posthoc Tukey, ##p < 0.0001, n = 23 images/genotype from 4 mice/genotype). **f** RIPA-insoluble fraction the cortex of 10-month-old CHCHD10^WT^, CHCHD10^R15L^, and CHCHD10^S59L^ immunoblotted for TDP-43 and actin. **g** Quantification of the insoluble TDP-43 normalized to actin from Fig. (2f) (1-way ANOVA, F(2, 6) = 17.13, P = 0.0033: posthoc Tukey, *p < 0.05, **p < 0.01, n = 3 mice/genotype)

To characterize pathologies in the CNS, we first examined the pattern of anti-CHCHD10 staining in the frontal cortex of 10-month-old WT, CHCHD10^WT^, CHCHD10^R15L^, and CHCHD10^S59L^ mice by immunohistochemistry (IHC). Immunofluorescence images showed that CHCHD10^R15L^ and CHCHD10^S59L^ mice frequently exhibit strong aggregate-like CHCHD10 staining in neuronal soma and punctuated staining in the neuropil (Fig. 2a). CHCHD10^WT^ and non-transgenic WT brains, however, exhibited largely punctuated CHCHD10 staining pattern both in the soma and neuropil (Fig. 2a; Additional file 2: Fig. S2d, control). Specifically, 19% of CHCHD10^R15L^ and 25% of CHCHD10^S59L^ expressing neurons in the frontal cortex exhibited strong CHCHD10 aggregate-like staining compared to < 5% in CHCHD10^WT^ containing neurons (Fig. 2b), despite more human Flag-CHCHD10 protein expression in CHCHD10^WT^ mice compared to CHCHD10^R15L^ and CHCHD10^S59L^ mice as seen by Western blotting (Additional file 2: Fig. S2e). Aggregate-like staining of CHCHD10 in CHCHD10^R15L^ and CHCHD10^S59L^ mice are reminiscent of the recently reported aggregation of the mouse CHCHD10^S55L^ knock-in mutant protein in vivo [33, 35].

To assess if CHCHD10 variants alter TDP-43, we next double stained brain sections for Flag-CHCHD10 and phospho-S409/410-TDP-43 [6, 36] as done in human brain tissues. Endogenous phospho-TDP-43 in the frontal cortex presented in a speckled irregular pattern in the cytoplasm (Fig. 2c, upper panels), which often colocalized with Flag-CHCHD10 variants in CHCHD10^R15L^ and CHCHD10^S59L^ mice (Fig. 2c, lower panels; Additional file 2: Fig. S2f, control). Phospho-TDP-43 immunoreactivity was significantly increased by 3.2- and 3.5-fold in CHCHD10^R15L^ and CHCHD10^S59L^ mouse frontal cortex, respectively, compared to CHCHD10^WT^ frontal cortex (Fig. 2c,d). The frequency of CHCHD10^S59L^ or CHCHD10^R15L^ colocalization with phospho-TDP-43 was 9- to 17-fold higher than that of Flag-CHCHD10^WT^ (Fig. 2e). As evidenced from phospho-TDP-43 staining, CHCHD10^R15L^ and CHCHD10^S59L^ brains exhibited 3.7- and 4.7-fold increases in RIPA-insoluble TDP-43, respectively, compared to CHCHD10^WT^ brains (Fig. 2f,g). Capture of large TDP-43 aggregates from sonicated RIPA-insoluble pellet by filter trap assay (FTA) revealed 1.7- and 1.8-fold increases in TDP-43 aggregates from CHCHD10^R15L^ and CHCHD10^S59L^ brains, respectively, compared to CHCHD10^WT^ brains (Additional file 2: Fig. S2g,h). These results were confirmed and extended in Tet-inducible Hela-myc-TDP-43 cells [37], where transient transfection of CHCHD10^R15L^ or CHCHD10^S59L^ resulted in both CHCHD10 and TDP-43 insolubility and aggregation compared to CHCHD10^WT^ transfection (Additional file 2: Fig. S2i-k).

### Long-term synaptic plasticity and motor unit deficits in CHCHD10^R15L^ and CHCHD10^S59L^ transgenic mice

**Fig. 3:**
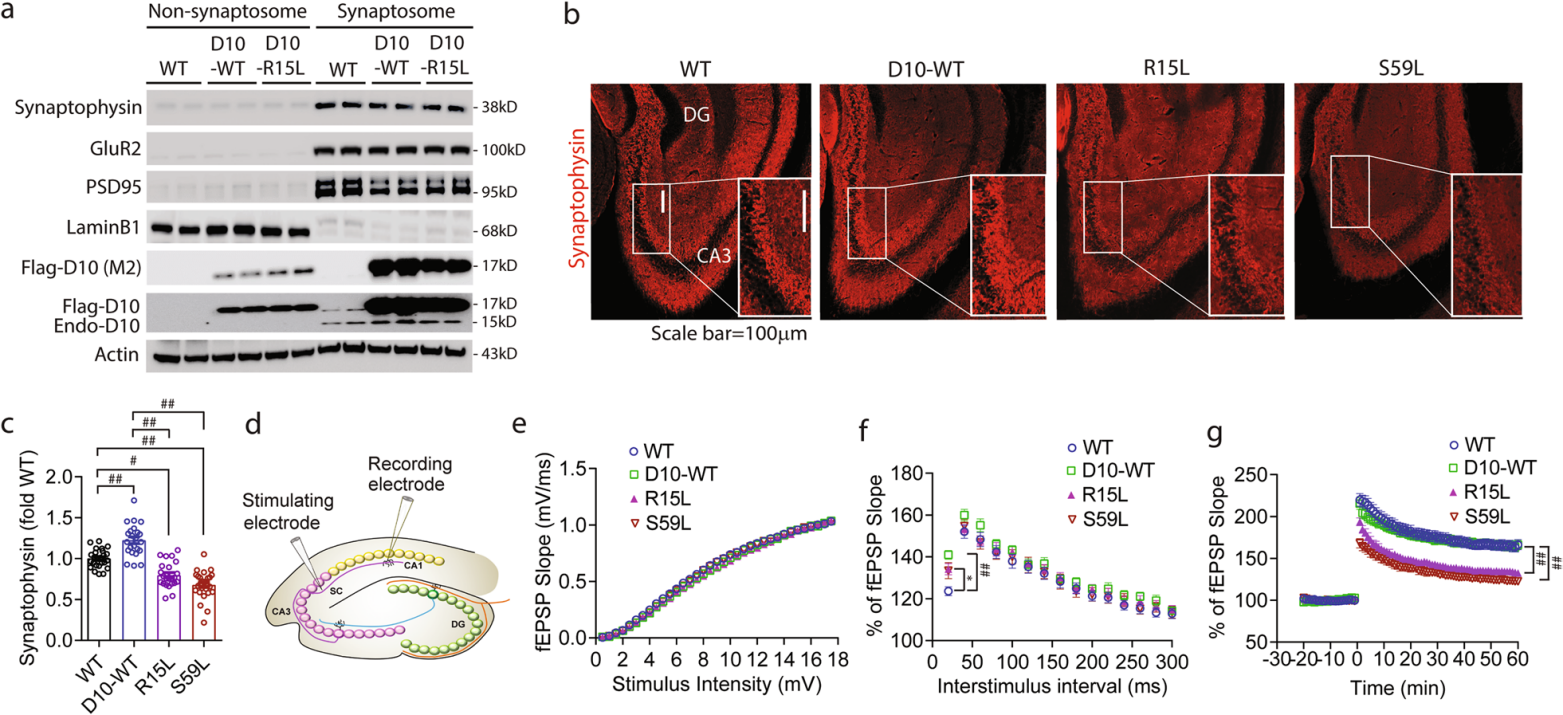
Long-term synaptic plasticity deficits in CHCHD10^R15L^ and CHCHD10^S59L^ transgenic mice. **a** Equal protein amounts subjected to biochemical fractionation for synaptosomes and cytosol from 10-month-old WT, CHCHD10^WT^, and CHCHD10^R15L^ mice and immunoblotted for the indicated proteins. **b** Representative images of brain sections from 10-month-old CHCHD10^WT^, CHCHD10^R15L^, and CHCHD10^S59L^ mice immunostained for synaptophysin (red). Small white boxes magnified in upper white boxed insets. **c** Quantification of synaptophysin intensity in hippocampus CA3 region from Fig. (3c) (1-way ANOVA – synaptophysin: F(3, 94) = 54.39, P < 0.0001: posthoc Tukey, *p < 0.05, #p < 0.001, ##p < 0.0001, n = 21–27 sections/genotype from 4 mice/genotype). **d** Schematic of short-term and long-term synaptic plasticity recordings from brain slices. **e-g** Brain slices from 10-month-old CHCHD10^WT^, CHCHD10^R15L^, and CHCHD10^S59L^ mice subjected to measurement of (e) input–output curve by stepping up stimulation amplitude from 0.5 to 18 mV (2-way ANOVA, genotype F(3, 4025) = 18.9, P < 0.0001: posthoc Tukey, not significant n = 25–32 slices/genotype from 4 mice/genotype), (f) PPF as a function of short-term plasticity from 20 to 300 ms interstimulus intervals (2-way ANOVA, genotype F(3, 1770) = 8.878, P < 0.0001: posthoc Tukey, *p < 0.05, ##p < 0.0001, n = 26–38 slices/genotype from 4 mice/genotype), and (g) LTP induced by theta burst stimulation (2-way ANOVA, genotype F(3, 9600) = 935.9, P < 0.0001: posthoc Tukey, ##p < 0.0001, n = 28 to 38 slices/genotype from 4 mice/genotype)

Mitochondria play vital roles in synaptic plasticity and are enriched at both sides of the synapse [38–40]. To assess whether CHCHD10 is biochemically enriched in synapses in vivo, we isolated synaptosome versus non-synaptosome fractions from brains of WT, CHCHD10^WT^, and CHCHD10^R15L^ mice. Both endogenous CHCHD10 and Flag-CHCHD10 variants were overwhelmingly localized to the synaptosome fraction enriched in synaptophysin, GluR2, and PSD95, despite equivalent levels of actin in the non-synaptosome fraction (Fig. 3a). As pathogenic animal models of TDP-43 demonstrate loss of synaptic integrity [17, 41, 42], we assessed synaptic integrity in 10-month-old CHCHD10 transgenic mouse variants and non-transgenic WT littermates by staining for synaptophysin. Examination of the CA3 region of the hippocampus showed that CHCHD10^R15L^ and CHCHD10^S59L^ brains exhibit significantly reduced immunoreactivity for synaptophysin compared to non-transgenic WT mice (Fig. 3b,c). In contrast, CHCHD10^WT^ mice showed significant increases in synaptophysin immunoreactivity compared to WT mice as well as CHCHD10^R15L^ and CHCHD10^S59L^ mice (Fig. 3b,c; Additional file 3: Fig. S3a, control). Assessment of astrogliosis by GFAP staining showed that CHCHD10^R15L^ and CHCHD10^S59L^ brains exhibit significantly increased GFAP immunoreactivity compared to CHCHD10^WT^ or WT mice at 10 months of age (Additional file 3: Fig. S3b,d). However, Iba1 staining to assess microgliosis did not reveal significant differences among WT mice and CHCHD10 variants (Additional file 3: Fig. S3c,e).

To determine functional changes in synaptic plasticity, we next subjected 10-month-old mouse acute brain slices to short-term and long-term synaptic plasticity measures, in which the stimulating electrode was placed in the Schaffer collaterals (SC) of the hippocampus and the recording electrode at the CA1 stratum radiatum below the pyramidal cell layer (Fig. 3d) [43]. Input–output (I-O) curves of the field excitatory postsynaptic potential (fEPSP) as a function of stepwise increase in stimulus intensity did not reveal significant differences among WT mice and CHCHD10 transgenic variants, indicating that basal synaptic transmission is not impacted by CHCHD10 transgenes (Fig. 3e). We next measured paired-pulse facilitation (PPF), a measure of presynaptic efficacy. While CHCHD10^WT^ mice exhibited significantly increased fEPSP compared to WT mice at the 20 ms interstimulus interval condition, no differences among genotypes (WT, CHCHD10^WT^, CHCHD10^R15L^, & CHCHD10^S59L^) were seen from 40 to 300 ms interstimulus intervals (Fig. 3f), indicating that CHCHD10 variants largely do not alter presynaptic facilitation. Finally, we measured basal fEPSP for 20 min followed by theta burst stimulation to elicit long-term potentiation (LTP). Both the induction and maintenance of LTP for 60 min were robust in WT and CHCHD10^WT^ slices, which did not differ from each other (Fig. 3g). However, CHCHD10^R15L^ and CHCHD10^S59L^ slices exhibited significant impairments in LTP throughout the course of 60 min compared to WT or CHCHD10^WT^ slices (Fig. 3g).

**Fig. 4:**
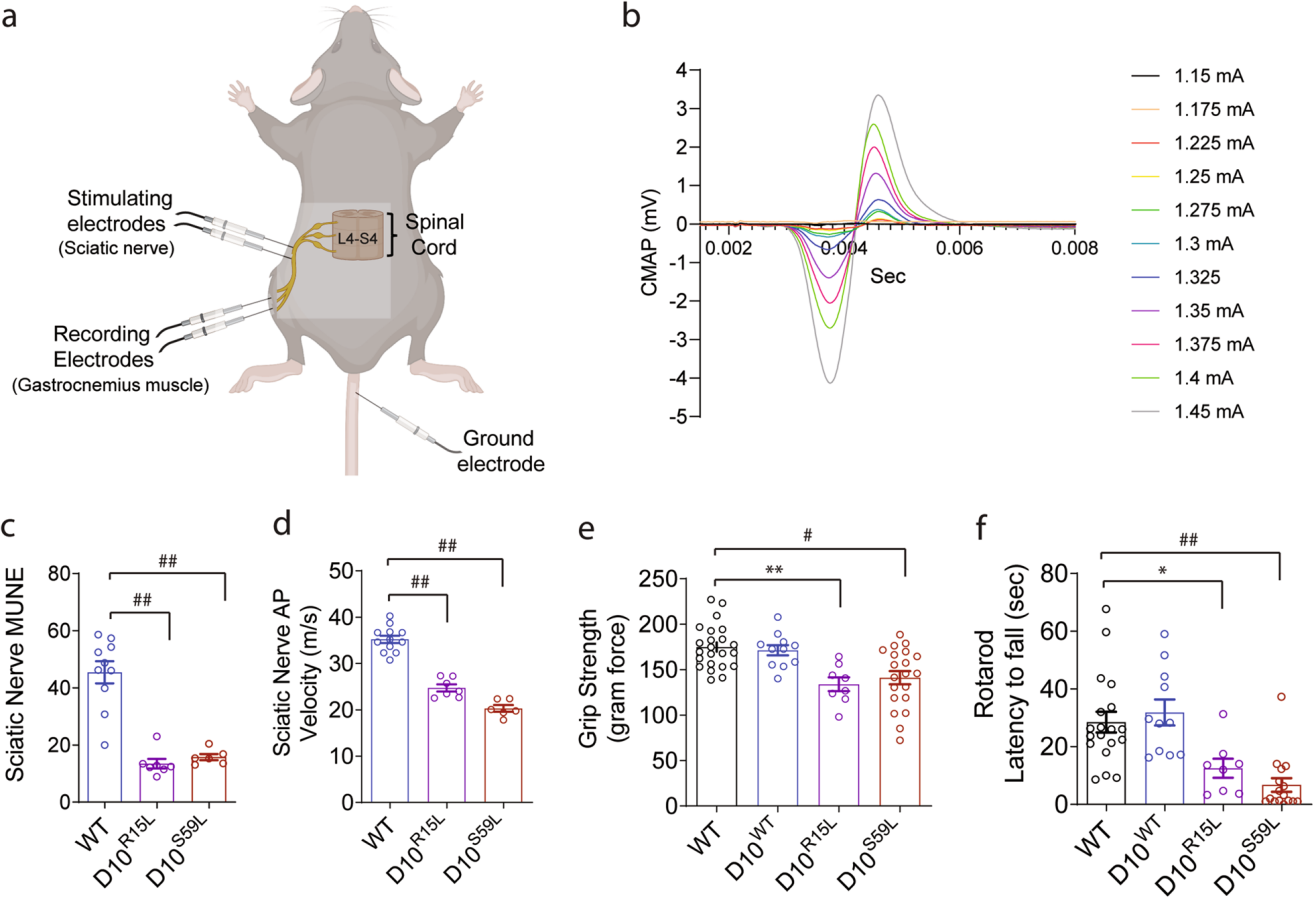
Functional motor unit and behavioral deficits in CHCHD10^R15L^ and CHCHD10^S59L^ transgenic mice. **a** Schematic of in vivo motor unit recording. Stimulating electrodes placed at the sciatic nerve and recording electrodes placed at the gastrocnemius muscle with grounding electrode at the tail. **b** A representative CMAP waveform from a 10-month-old wild type mouse. Stimulus intensity at the sciatic nerve was increased at increments of 0.025 mA until compound muscle action potential (CMAP) was evoked in an all-or-none fashion and repeated for a total of 10 increments to calculate single motor unit potential (SMUP) and motor unit number estimation (MUNE). **c** Motor unit number estimation (MUNE) of 10-month-old WT, CHCHD10^WT^, CHCHD10^R15L^, and CHCHD10^S59L^ mice (1-way ANOVA, F(2, 20) = 36, P < 0.0001, posthoc Tukey, ##P < 0.0001, n = 6–10 mice/genotype). **d** Sciatic nerve CMAP velocity (m/s) measured between the stimulating and recording electrodes in 10-month-old WT, CHCHD10^WT^, CHCHD10^R15L^, and CHCHD10^S59L^ mice (1-way ANOVA, F(2, 22) = 91.14, P < 0.0001, posthoc Tukey, ##P < 0.0001, n = 6–12 mice/genotype). **e** Grip strength (gram force) in 10-month-old WT, CHCHD10^WT^, CHCHD10^R15L^, and CHCHD10^S59L^ mice (1-way ANOVA, F(3, 58) = 9.072, P < 0.0001, posthoc Tukey, **P < 0.01, #P < 0.001, n = 8–23 mice/genotype). **f** Rotarod test measured in latency to fall (sec) in 10-month-old WT, CHCHD10^WT^, CHCHD10^R15L^, and CHCHD10^S59L^ mice (1-way ANOVA, F(3, 50) = 12, P < 0.0001, posthoc Tukey, *P < 0.05, ##P < 0.0001, n = 8–19 mice/genotype)

As CHCHD10 mutations are associated with the spectrum of FTD and ALS, we next assessed electrophysiological measures of motor neurons innervating the muscle via the sciatic nerve, a spinal nerve that carries motor neuron outputs through the sacral plexus (L4-S4) and innervates most of the hind limb. In 10-month-old mice, we carried out in vivo recordings at the gastrocnemius muscle after stimulation of the sciatic nerve using the incremental motor unit number estimation (MUNE) method (Fig. 4a), which allows for a functional readout of motor neuron connectivity with the muscle [44, 45]. Stimulus intensity at the sciatic nerve was increased at increments of 0.025 mA until compound muscle action potential (CMAP) was evoked in an all-or-none fashion and repeated for a total of 10 increments (Fig. 4b) to calculate single motor unit potential (SMUP) and MUNE, a sensitive measure to identify and track motor unit loss [44–46]. Ten-month-old CHCHD10^R15L^ and CHCHD10^S59L^ mice exhibited severely reduced MUNE (Fig. 4c), indicative of motor unit loss [44, 45]. In addition, CHCHD10^R15L^ and CHCHD10^S59L^ mice also exhibited significantly reduced sciatic nerve compound action potential velocity (Fig. 4d), indicative of axonal pathology. Accordingly, 10-month-old CHCHD10^R15L^ and CHCHD10^S59L^ mice exhibited significant deficits in grip strength (Fig. 4e) and shortened latency to fall from rotarod (Fig. 4f) compared to WT and CHCHD10^WT^ mice. Such electrophysiological and behavioral deficits were consistent with expression of CHCHD10 variants in ChAT-positive motor neurons of the lumbar spinal cord (Additional file 4: Fig. S4a) and significantly higher phospho-S409/S410-TDP-43 immunoreactivity in spinal motor neurons of CHCHD10^R15L^ and CHCHD10^S59L^ mice (> twofold) compared to WT and CHCHD10^WT^ mice (Additional file 4: Fig. S4b,c).

### CHCHD10.^WT^ mitigates TDP-43 pathology and rescues synaptic plasticity deficits in TDP-43 transgenic mice

**Fig. 5:**
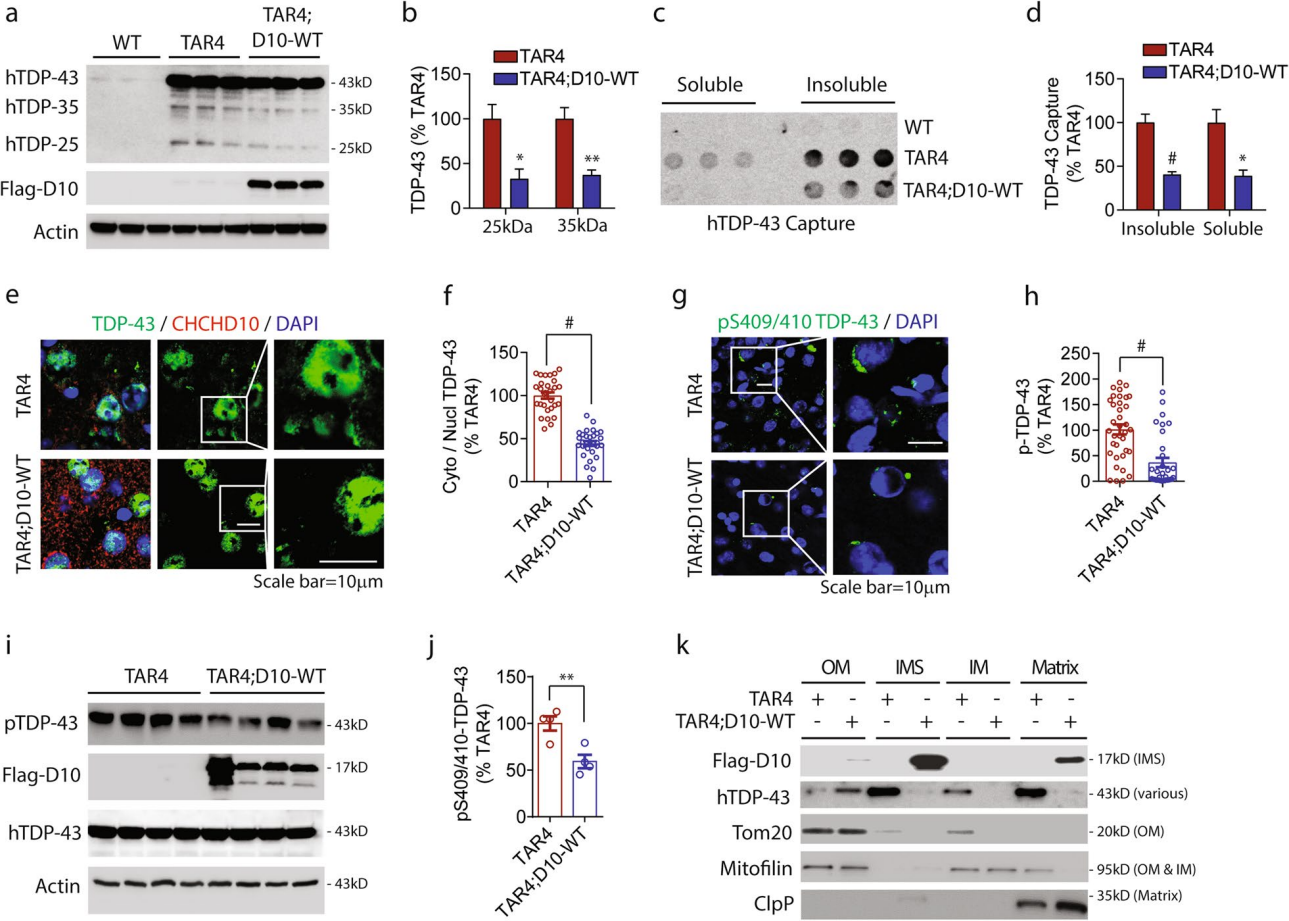
CHCHD10^WT^ mitigates TDP-43 pathology in TDP-43 transgenic mice (TAR4). **a** Equal protein amounts of RIPA-soluble extracts from cortex of 10-month old WT, TAR4, and TAR4;D10^WT^ mice immunoblotted for human TDP-43 (hTDP-43), Flag-CHCHD10 (Flag-M2), and actin. **b** Quantification of 25-kDa and 35-kDa hTDP-43 C-terminal fragments from Fig. (5a) (t-test, *p < 0.05, **p < 0.01, n = 3 mice/genotype). **c** Equal protein amounts of RIPA-soluble and sonicated RIPA-insoluble extracts from the cortex of WT, TAR4, and TAR4;D10^WT^ mice subjected to filter trap assay for hTDP-43. **d** Quantification of hTDP-43 captured by filter-trap assay from Fig. (5c) (t-test, *p < 0.05, #p < 0.001, n = 3 mice/genotype). **e** Representative images of brain sections from 10-month-old TAR4 and TAR4;D10^WT^ mice immunostained for hTDP-43 (green), CHCHD10 (red) and DAPI (blue). White boxes magnified in right panels. **f** Quantification of cytoplasmic to nuclear TDP-43 intensity ratio from Fig. (5e) (t-test, #p < 0.001, n = 29–30 sections/genotype from 4 mice/genotype). **g** Representative images of brain sections from 10-month-old TAR4 and TAR4;D10^WT^ mice immunostained for human pS409/410-TDP-43 (green) and DAPI (blue). White boxes magnified in right panels. **h** Quantification of pS409/410-TDP-43 intensity from Fig. (5 g) (t-test, #p < 0.001, n = 33–36 sections/genotype from 4 mice/genotype). **i** Equal protein amounts of RIPA extracts from cortex of 10-month old TAR4 and TAR4;D10^WT^ mice immunoblotted for pS409/410-TDP-43, total hTDP-43, Flag-CHCHD10 (M2), and actin. **j** Quantification of pS409/410-TDP-43 from Fig. (5i) (t-test, **p < 0.01, n = 4 mice/genotype). **k** Mitochondria isolated from 10-month-old TAR4 and TAR4;D10^WT^ mouse brains sub-fractionated for outer membrane (OM), intermembrane space (IMS), inner membrane (IM) and matrix and then immunoblotted for hTDP-43, Flag-CHCHD10 (M2), Tom20, mitofilin, and ClpP. Known sub-mitochondrial localization of indicated proteins in parenthesis

Human FTLD-TDP patients and TDP-43 transgenic mice (TAR4) exhibit significantly reduced level of soluble CHCHD10 protein [26], suggesting that loss of functional CHCHD10 could contribute to TDP-43 pathogenesis. To test the hypothesis that restoring CHCHD10 expression in TDP-43 transgenic mice (TAR4) mitigates TDP-43 pathogenesis in vivo, we crossed hemizygous TAR4 mice expressing wild type human TDP-43 driven by the *Thy-1* promoter [29] with hemizygous CHCHD10^WT^ transgenic mice to generate WT, TAR4, and TAR4;CHCHD10^WT^ (TAR4;D10^WT^) littermates. At 10 months of age, TAR4;D10^WT^ mice did not alter full-length human 43-kDa TDP-43 levels in the RIPA-soluble fraction compared to littermate TAR4 mice, although significant ~ 60% reductions in both the 25-kDa and 35-kDa TDP-43 fragments were observed in TAR4;D10^WT^ mice compared to TAR4 mice (Fig. 5a,b). As these C-terminal 25-kDa and 35-kDa fragments are more insoluble and prone to aggregation [47–49], we performed filter-trap assays (FTA) using RIPA-soluble and sonicated RIPA-insoluble brain extracts from WT, TAR4, and TAR4;D10^WT^ littermates. TDP-43 aggregates captured using a human TDP-43-specific antibody in FTA was significantly reduced by ~ 60% in TAR4;D10^WT^ versus TAR4 extracts in both RIPA-soluble and RIPA-insoluble fractions (Fig. 5c,d). As expected, no human TDP-43 aggregates were captured in WT littermate brain extracts (Fig. 5c). Staining for human TDP-43 demonstrated largely nuclear localization but also some cytoplasmic mislocalization in TAR4 mice (Fig. 5e), the latter which was significantly reduced by > 50% in TAR4;D10^WT^ frontal cortex (Fig. 5e,f; Additional file 5: Fig. S5a, control) and TAR4;D10^WT^ lumbar spinal cords compared to those of TAR4 mice (Additional file 5: Fig. S5c,d). We also stained for phospho-S409/S410-TDP-43, which was localized to the cytoplasm of TAR4 mice both in the frontal cortex (Fig. 5g; Additional file 5: Fig. S5b, control) and lumbar spinal cords (Additional file 5: Fig. S5e,f). Phospho-TDP-43 staining was significantly reduced by 64% in TAR4;D10^WT^ frontal cortex (Fig. 5g,h) and ~ 50% in TAR4;D10^WT^ lumbar spinal cord (Additional file 5: Fig. S5e,f) compared to those in TAR4 mice. Consistent with phospho-TDP-43 staining, Western blotting of cortical brain extracts confirmed the significant reduction of phospho-TDP-43 in TAR4;D10^WT^ mice compared to TAR4 littermates (Fig. 5i,j). To determine if CHCHD10 alters the sub-mitochondrial accumulation of TDP-43, we isolated mitochondria from TAR4 and TAR4;D10^WT^ mice and fractionated the outer membrane (OM), intermembrane space, (IMS), inner membrane (IM), and matrix. As shown in preceding results, Flag-CHCHD10^WT^ was highly enriched in the IMS [50] with minor localization in the matrix (Fig. 5k). TDP-43 was equally enriched in the IMS and matrix in TAR4 mitochondria (Fig. 5k). In TAR4;D10^WT^ mitochondria, however, TDP-43 was generally reduced in mitochondria and absent in fractions where Flag-CHCHD10^WT^ was most enriched (IMS & matrix) (Fig. 5k), consistent with our preceding findings. These results collectively demonstrate that restoring or elevating CHCHD10^WT^ expression mitigates TDP-43 pathology in vivo.

**Fig. 6:**
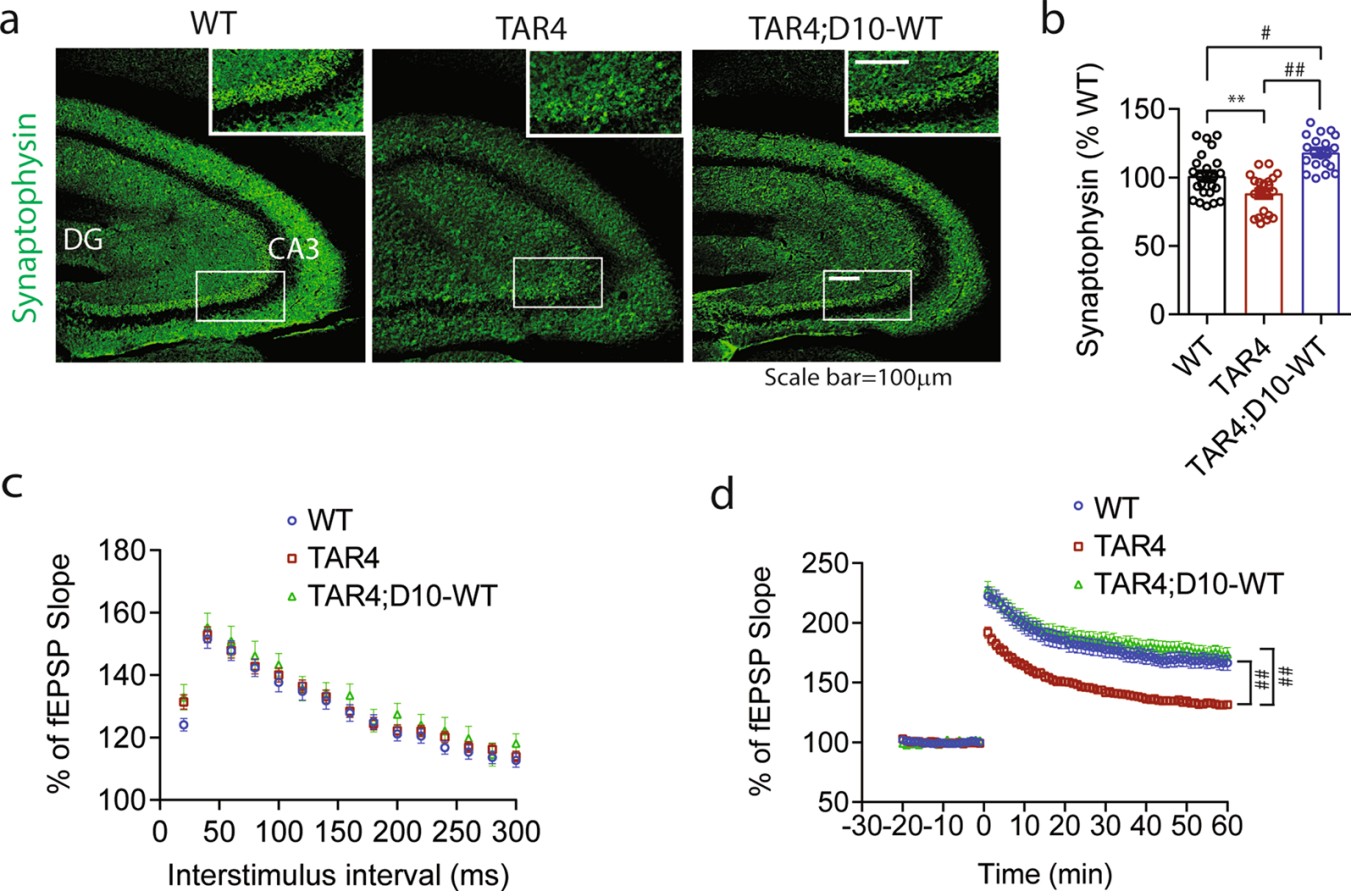
CHCHD10^WT^ rescues impaired synaptic plasticity in TDP-43 transgenic mice (TAR4). **a** Representative brain section images from 10-month-old TAR4 and TAR4;D10^WT^ mice immunostained for synaptophysin (green). Small white boxes magnified in larger upper right insets. **b** Quantification of synaptophysin intensity in hippocampus CA3 region from Fig. (6a) (1-way ANOVA – synaptophysin: F(2, 63) = 23.07, P < 0.0001; posthoc Tukey, **p < 0.01, #p < 0.001, ##p < 0.0001, n = 20–27 section/genotype from 4 mice/genotype). **c-d** Brain slices from 10-month-old TAR4 and TAR4;D10^WT^ mice subjected to (c) PPF from 20 to 300 ms interstimulus intervals (2-way ANOVA, genotype F(2, 1815) = 7.891, P = 0.0004: posthoc Tukey, not significant, n = 24–58 slices from 3–5 mice/genotype) and (d) LTP induced by theta burst stimulation (2-way ANOVA, genotype F(2, 8640) = 991.0, P < 0.0001: posthoc Tukey, ##p < 0.0001, n = 24–51 slices/genotype from 3–5 mice/genotype)

Finally, we examined the functional role of CHCHD10 on synaptic integrity and synaptic plasticity in 10-month-old WT, TAR4, and TAR4;10^WT^ littermates. Hemizygous TAR4 mice exhibited significantly reduced immunoreactivity for synaptic markers synaptophysin in the CA3 region of the hippocampus compared to WT mice, which was completely rescued to or beyond WT levels in TAR4;D10^WT^ mice (Fig. 6a,b). In acute brain slice recordings from 10-month-old littermate mice, we observed little to no alterations in input–output (I-O) curves among the genotypes (Additional file 6: Fig. S6). Although no significance differences were detected in PPF among the genotypes, TAR4;D10^WT^ mice tended to exhibit higher fEPSP slope than WT or TAR4 mice at all interstimulus intervals (Fig. 6c). TAR4 brain slices exhibited significantly impaired LTP after theta burst stimulation compared to WT mice at all time points (Fig. 6d). Such impairment in LTP was completely rescued by CHCHD10 expression in TAR4;D10^WT^ mice to levels indistinguishable from WT slices (Fig. 6d), consistent with the protective effects of CHCHD10^WT^ against TDP-43 pathogenesis.

### Mutant CHCHD10 promotes mitochondrial TDP-43 accumulation and aggregation

**Fig. 7:**
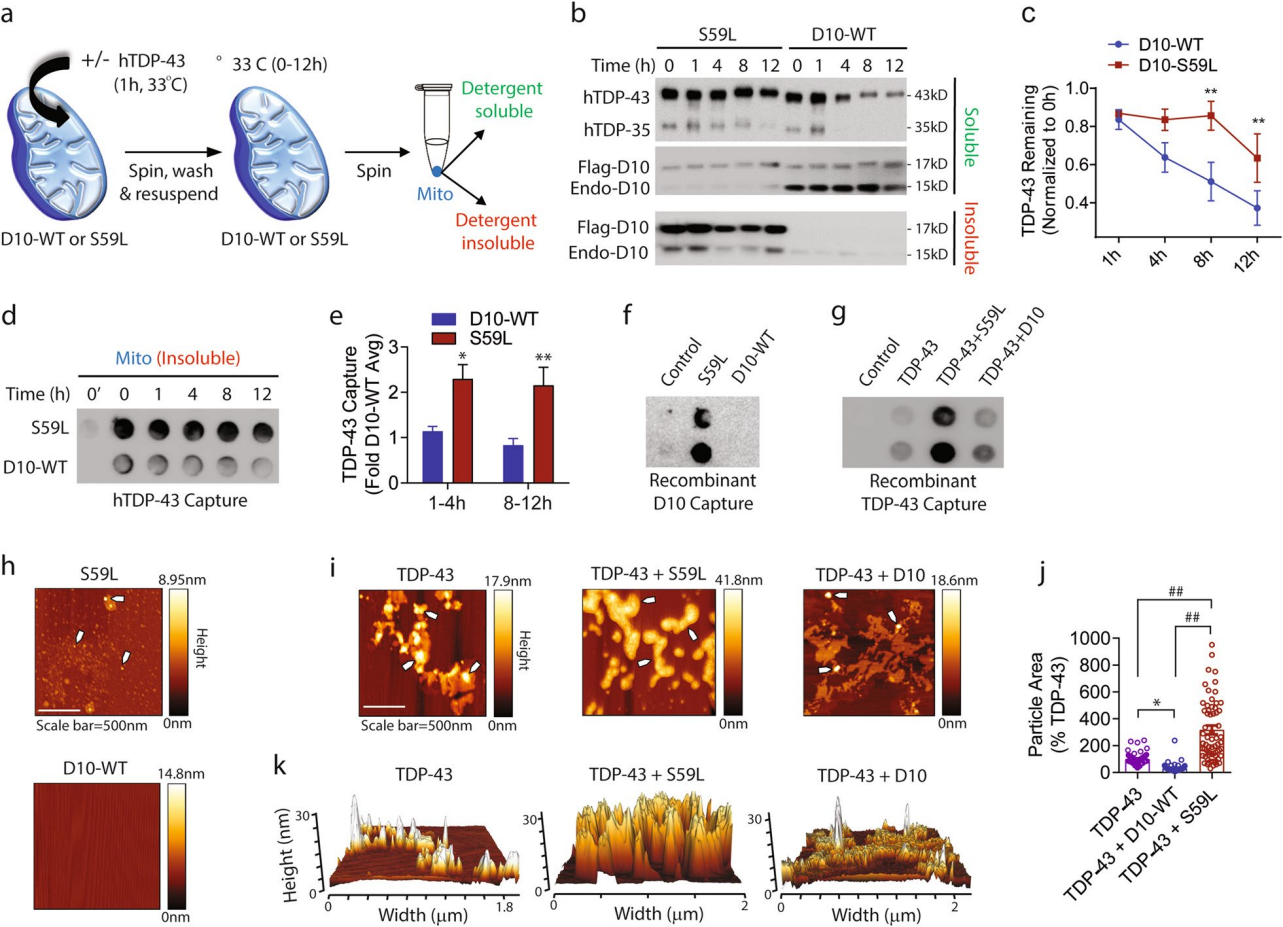
Mutant CHCHD10 promotes mitochondrial TDP-43 aggregation and increases the size of TDP-43 aggregates in vitro. **a** Schematic of hTDP-43 mitochondria import and turnover assay for (**b–e**). HEK293T cells were transfected with Flag-CHCHD10^WT^ or Flag-CHCHD10^S59L^ and subjected to isolated of mitochondria. Human recombinant TDP-43 (hTDP-43) protein was imported into isolated mitochondria by incubation for 1 h at 33 °C, spun & washed, and chased for 0–12 h at 33 °C, after which mitochondria were lysed with RIPA to separate RIPA-soluble and RIPA-insoluble fractions. **b** Representative blots showing equal protein amounts of RIPA-soluble and RIPA-insoluble mitochondria immunoblotted for hTDP-43 and CHCHD10. **c** Quantification of recombinant hTDP-43 remaining in mitochondria over 12 h from RIPA-soluble fractions from Fig. (2b) (2-way ANOVA, group F(1, 3) = 9.69, P = 0.05: posthoc Sidak, *p < 0.05, **p < 0.01, n = 4/group). **d** Equal amounts of sonicated RIPA-insoluble fractions subjected to filter trap assay for hTDP-43. **e** Quantification of filter-trapped hTDP-43 aggregates from Fig. 2d classified by 1-4 h and 8-12 h time points (2-way ANOVA, group F(1, 8) = 12.07, P = 0.0084: posthoc Sidak, *p < 0.05, **p < 0.01, n = 4/group). **f** Recombinant CHCHD10^WT^ or CHCHD10^S59L^ protein incubated alone with vigorous shaking at 37 °C for 8 h and subjected to filter trap assay for CHCHD10. **g** Recombinant TDP-43 protein incubated alone or co-incubated with CHCHD10^WT^ or CHCHD10.^S59L^ protein with vigorous shaking at 37 °C for 8 h and subjected to filter trap assay for TDP-43. **h–i** Representative AFM images of recombinant proteins from Fig. 2f and g. Maximal particle height indicated by color-coded side bars. Arrows indicate relatively high-height protein particles per side bar scale. **j** Quantification of protein particle area among particles > 10 nm in height (1-way ANOVA, F(2, 182) = 51.36, P < 0.0001: posthoc Tukey, *p < 0.05, ##p < 0.0001, n = 57-67 particles/group). **k** Representative 3-dimensional reconstruction of AFM images showing particle contour, height, and area

TDP-43 is readily imported into mitochondria [16] and misfolded or aggregation-prone proteins in the cytosol are frequently imported into mitochondria to be degraded, which otherwise could overwhelm the cytosolic proteostasis machinery [51]. As CHCHD10 is a mitochondria-resident protein localized to the intermembrane space [50] and physically interacts with TDP-43 [17], we hypothesized that FTD/ALS-linked CHCHD10 mutations promote the accumulation of misfolded TDP-43 upon mislocalization to the cytoplasm and import into mitochondria. To test the hypothesis, we isolated intact mitochondria from HEK293T cells transfected with Flag-CHCHD10^WT^ or Flag-CHCHD10^S59L^. We then exposed mitochondria to human recombinant TDP-43 to perform mitochondrial import for 1 h at 33 °C, after which mitochondria were spun to remove unimported hTDP-43 (Fig. 7a). Resuspended mitochondria were then returned to 33 °C for 0-12 h. After each time point, mitochondria were collected by centrifugation, lysed with RIPA, and soluble supernatant and insoluble pellet were collected (Fig. 7a). Each fraction was then subjected to Western blotting and/or filter-trap assay (FTA). Western blotting for imported TDP-43 showed that CHCHD10^S59L^-containing mitochondria exhibit significantly slower turnover of TDP-43 over 12 h compared to CHCHD10^WT^ containing mitochondria (Fig. 7b,c). Interestingly, the C-terminal 35-kDa fragment of TDP-43 was also detected in mitochondria, which exhibited similar turnover rates as full-length TDP-43 (Fig. 7b). This was accompanied by the expected accumulation of insoluble CHCHD10^S59L^ versus CHCHD10^WT^ in the pellet fraction (Fig. 7b, lower panel) and significantly increased capture of TDP-43 aggregates in CHCHD10^S59L^-containing insoluble mitochondrial pellet by FTA (Fig. 7d,e). This insoluble aggregated pool of TDP-43 was seen early after import and was relatively stable over the 12 h period (Fig. 7d,e), which likely accounts for the slower turnover of imported TDP-43 in CHCHD10^S59L^-containing mitochondria. Little to no TDP-43 was captured in the absence of recombinant TDP-43 import (Fig. 7d: time 0’), demonstrating the specificity of the FTA. In support of these findings, biochemically isolated mitochondria from CHCHD10^R15L^ and CHCHD10^S59L^ mouse brains exhibited significantly more endogenous TDP-43 compared to mitochondria from CHCHD10^WT^ mouse brains (Additional file 7: Fig S7a,b). Furthermore, in CHCHD10 transgenic primary neurons transduced with TDP-43-tomato AAV9, CHCHD10^R15L^ and CHCHD10^S59L^ neurons demonstrated significantly increased TDP-43 cytoplasmic mislocalization and colocalization with the mitochondrial marker Tom20 than CHCHD10^WT^ and WT neurons (Additional file 7: Fig. S7c, d).

### Mutant CHCHD10 promotes and wild type CHCHD10 inhibits TDP-43 aggregation in vitro

As CHCHD10 binds to TDP-43 [17] and CHCHD10^S59L^ blocks TDP-43 turnover and promotes its aggregation upon import into mitochondria, we tested if CHCHD10^WT^ or CHCHD10^S59L^ can directly alter TDP-43 aggregation using purified recombinant proteins. We purified His-tagged CHCHD10^WT^ and CHCHD10^S59L^ from *E. Coli* (Additional file 8: Fig. S8) and purchased human recombinant TDP-43 (R&D Systems). We incubated purified CHCHD10 variants or TDP-43 by themselves (each at 1 μM) or co-incubated TDP-43 (1 μM) with CHCHD10 variants (1 μM) at 37 °C for 8 h with vigorous shaking, after which samples were subjected to filter trap assay (FTA) and atomic force microscopy (AFM) (Supplemental methods). When incubated alone, CHCHD10^S59L^ (Fig. 7f) and TDP-43 (Fig. 7g) protein aggregates were detected by FTA, whereas no CHCHD10^WT^ protein aggregates were detected (Fig. 7f). Co-incubation of TDP-43 with CHCHD10^S59L^ but not CHCHD10^WT^ strongly increased TDP-43 aggregation as detected by FTA (Fig. 7g). Visualization of protein aggregates by AFM showed the presence of small oligomer-like CHCHD10^S59L^ aggregates but little to no CHCHD10^WT^ aggregates when incubated alone (Fig. 7h). TDP-43 alone formed high-contrast aggregates indicative of increased particle height, which appeared to arise from the low-contrast material of low particle height (Fig. 7i, left panel). Co-incubation of TDP-43 with CHCHD10^S59L^ altered the morphology of the particles, such that the high-height particles significantly increased in area (large spheroid morphology) at the expense of the low-height material (Fig. 7i, middle panel), reminiscent of the buoyant FTLD-TDP ‘type A’ aggregates [52]. Co-incubation of TDP-43 with CHCHD10^WT^, however, significantly altered by morphology of protein particles by decreasing high-height particles in favor of low-height material (Fig. 7i, right panel). Quantification of high-height particles > 10 nm showed that CHCHD10^S59L^ significantly increases the area of high-height particle by > threefold, whereas CHCHD10^WT^ significantly decreases the areas of high-height particle by > 50% (Fig. 7j), clearly indicating that CHCHD10^S59L^ increases the size of aggregates whereas CHCHD10^WT^ reduces the growth of aggregates. Three-dimensional reconstruction of representative AFM images illustrates the typical height and area of the particles corresponding to each condition (Fig. 7k). These results taken together indicate that CHCHD10^S59L^ increases TDP-43 aggregation upon import into mitochondria, likely by directly co-aggregating with TDP-43, whereas the direct interaction of CHCHD10^WT^ with TDP-43 inhibits TDP-43 aggregation.

## Discussion

TDP-43 inclusions are pathologically evident in more than 95% of ALS, ~ 50% of FTD (FTLD-TDP variant), and > 50% of late-onset AD [4, 8, 9]. As our earlier study found that detergent soluble CHCHD10 levels significantly decline in brains of FTLD-TDP patients and TDP-43 transgenic mice, we initially looked for changes in CHCHD10 staining together with phospho-TDP-43 staining in brains of FTLD-TDP, AD, and nondemented controls. Despite the depletion of CHCHD10 cytoplasmic neuronal staining in FTLD-TDP, we encountered the surprising finding that insoluble CHCHD10 aggregates accumulate and colocalize with phospho-TDP43 inclusions in FTLD-TDP and AD. Such intriguing pathological correlation in post-mortem human brains called for a careful examination of the mechanistic connection between CHCHD10 and TDP-43 pathologies utilizing experimental animal models. We showed that CHCHD10^R15L^ and CHCHD10^S59L^ mice exhibit CHCHD10 aggregates associated with elevated phospho-TDP-43 in vivo. By Western blotting and filter trap assays, we found increased insoluble CHCHD10 and TDP-43 aggregates induced by CHCHD10^R15L^ and CHCHD10^S59L^ mutations. Such proteopathic phenotypes were consistent with pathogenesis originating from mitochondria, as both primary neurons and brains derived from CHCHD10^R15L^ and CHCHD10^S59L^ mice showed mitochondrial accumulation of TDP-43. Isolated mitochondria expressing CHCHD10^S59L^ exhibited impaired proteostasis of imported TDP-43 as evidenced by enhanced aggregation of both proteins. Using purified recombinant proteins, we showed that CHCHD10^S59L^ directly increases TDP-43 aggregate size, whereas CHCHD10^WT^ inhibits TDP-43 aggregation. These changes mirrored the functional impairments in long-term synaptic plasticity in brain as well as deficits in motor neuron unit function, sciatic nerve action potential velocity, grip strength, and rotarod performance seen in CHCHD10^R15L^ and CHCHD10^S59L^ mice. As endogenous soluble CHCHD10 levels decline significantly in brains of FTLD-TDP patients and TDP-43 transgenic mice [26] and CHCHD10^WT^ inhibits TDP-43 aggregation in vitro, we also showed that restoring CHCHD10^WT^ in TDP-43 transgenic mice (TAR4;D10^WT^) mitigates TDP-43 pathology and rescues TDP-43-induced deficits in long-term synaptic plasticity in vivo. These results overall implicate CHCHD10-mediated modulation of TDP-43 aggregation in mitochondria as a significant factor contributing to impairments in long-term synaptic plasticity and motor unit function.

Mitochondrial targeting of misfolded or aggregation-prone proteins from the cytosol has recently been proposed as a pathway to handle and turnover misfolded proteins within mitochondria, which otherwise would overwhelm the cytosolic proteostasis machinery [51]. This mitochondria-mediated proteostasis mechanism was initially discovered in yeast and named ‘mitochondria as guardian in cytosol’ (MAGIC) [51]. While previous studies have found TDP-43 to be translocated into mitochondria with ALS-linked TDP-43 mutations exhibiting greater translocation efficiency [16], this study for the first time showed that TDP-43 is subject to CHCHD10-regulated aggregation and turnover upon import into mitochondria. Our observation that isolated mitochondria containing CHCHD10^S59L^ significantly increases TDP-43 aggregation upon import is consistent with the accumulation of TDP-43 in mitochondria in CHCHD10^R15L^ and CHCHD10^S59L^ transgenic primary neurons and brains in vivo. These results are also consistent with a recent study showing that the CHCHD10^S59L^ mutation increases insoluble mitochondrial TDP-43 in transfected HeLa cells [53]. While the authors interpreted this to indicate increased mitochondrial translocation of TDP-43 [53], our results instead indicate that CHCHD10 mutations enhance the aggregation of TDP-43 upon its translocation into mitochondria, likely via co-aggregation of both proteins. This is an important distinction, as our mechanistic model predicts that sufficient mitochondrial translocation of TDP-43 is a necessary secondary trigger allowing mutant or misfolded CHCHD10 to exacerbate TDP-43 pathology. It is notable that TDP-43 inclusions were not observed in the only autopsied patient carrying a *CHCHD10* mutation to date (p.R15L) [54]. In this patient, lack of the TDP-43 pathology component due to insufficient trigger of mitochondrial TDP-43 translocation may underlie the very slow progression of disease (19 years to death) and atypical ALS symptoms [54]. In this light, it would be highly informative to correlate disease progression with the severity of TDP-43 pathology in future autopsy studies of FTD-ALS patients carrying *CHCHD10* mutations. Our findings from FTLD-TDP and AD cases showing robust colocalization phospho-TDP-43 pathology with CHCHD10 inclusions (~ 75–84%) and tight correlation between insoluble CHCHD10 with insoluble TDP-43 indeed indicates a pathological link between them in human neurodegenerative diseases. Beyond TDP-43-related spectrum disorders, our mechanistic model may also help to shed light on the diversity of disease phenotypes associated with *CHCHD10* mutations, such as mitochondrial myopathy [55], spinal muscular atrophy (SMA) [56], and Charcot-Marie-Tooth disease type 2 (CMT2) [57]. In such cases, secondary triggers, if any, may represent different aggregation-prone proteins expressed in disease-relevant tissues that may also be subject to CHCHD10-regulated proteostasis and aggregation.

**Fig. 8:**
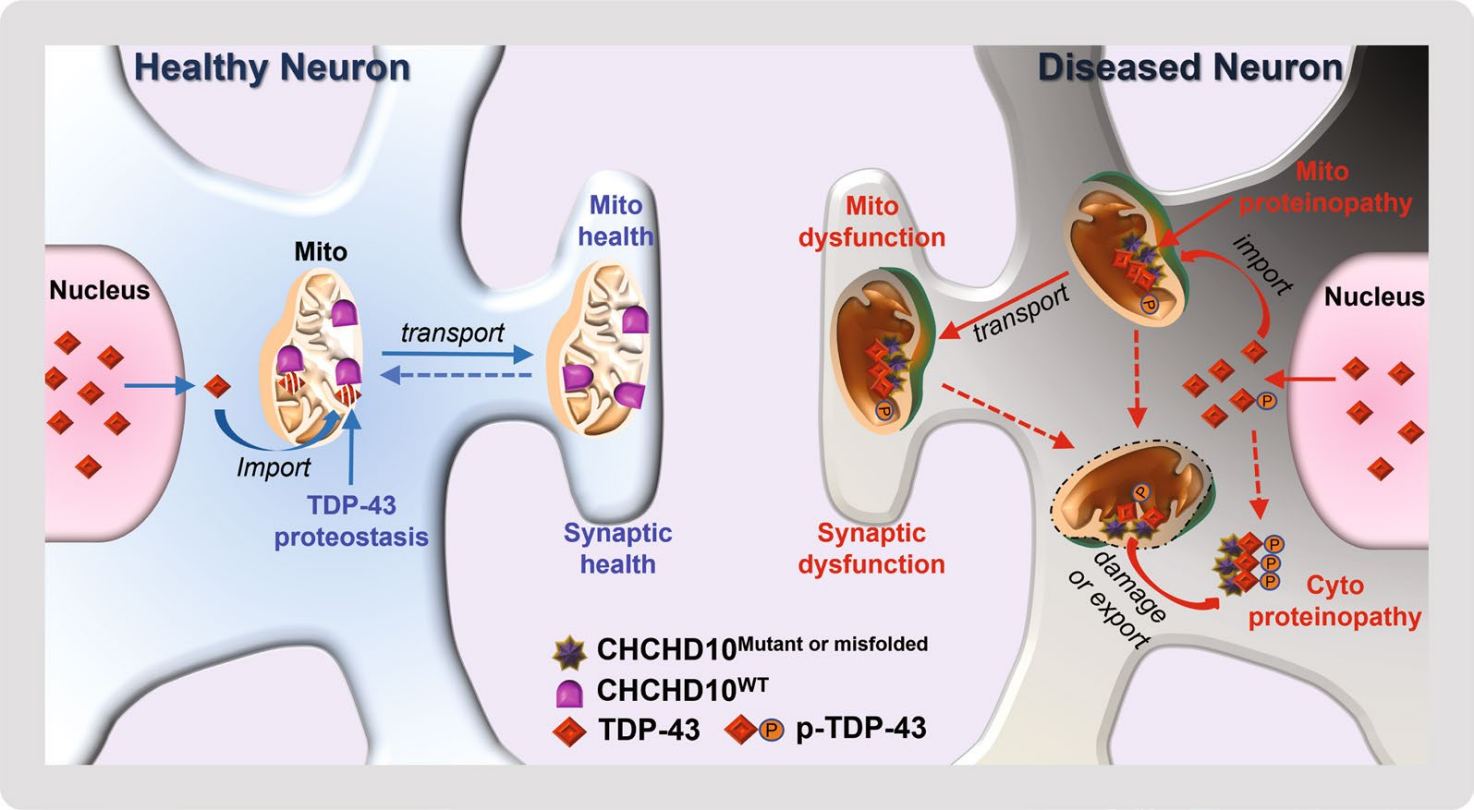
Schematic model of CHCHD10 in mitochondrial proteinopathy and synaptic function. In healthy neurons, small amounts of misfolded proteins, such as TDP-43, accumulate in the cytoplasm, some of which are imported into mitochondria. Mitochondria containing a healthy level of soluble CHCHD10^WT^ inhibits TDP-43 insolubility and aggregation, thereby sustaining mitochondrial health. Healthy mitochondria at the synapse facilitates long-term synaptic plasticity and motor unit function. In FTLD-TDP brains, soluble CHCHD10 declines and instead deposits as insoluble CHCHD10 aggregates with phospho-TDP-43. Mechanistically, in diseased neurons, excessive amounts of misfolded proteins, such as TDP-43 or phospho-TDP-43, accumulate in the cytoplasm, some of which are imported into mitochondria. Mitochondria containing mutant and/or misfolded CHCHD10 enhance both CHCHD10 and TDP-43 aggregation, thereby driving mitochondrial proteinopathy and dysfunction. Aggregate-harboring mitochondria may contribute to cytoplasmic inclusions upon mitochondrial damage and release of the aggregate content to the cytosol. At the synapse, proteopathic mitochondria become dysfunctional and fail to support long-term synaptic plasticity or motor unit function

The propensity for mutant CHCHD10 to misfold and aggregate per se represents a gain-of-function molecular phenotypes intrinsic to its protein structure [33, 35]. We speculate that such misfolding could also be induced in CHCHD10^WT^ under pathological conditions, perhaps through excessive mitochondrial translocation of TDP-43 (i.e. FTLD-TDP or AD). In this scenario, the loss of functional CHCHD10 or gain of misfolded CHCHD10 fails to oppose or directly promotes TDP-43 aggregation, respectively, thereby generating a maladaptive feed-forward cycle of mitochondrial proteinopathy. This notion is supported by the observation that insoluble TDP-43 positively correlates with insoluble CHCHD10 in human brains. Accumulation of TDP-43 and mutant CHCHD10 in mitochondria are damaging to mitochondria and neurons that house them [17, 26], and blockade of mitochondrial TDP-43 translocation also significantly attenuates CHCHD10^S^^59^^L^-mediated toxicity [53]. Hence, aggregate-harboring mitochondria likely also contribute to cytosolic inclusions upon mitochondrial damage and release of the aggregate content to the cytosol (Fig. 8).

The CHCHD10^R15L^ and CHCHD10^S59L^ transgenic models used in this study faithfully recapitulated many pathological phenotypes associated with FTD-ALS. These included CHCHD10 pathology and increased pTDP-43 levels associated with deficits in synaptic integrity, long-term synaptic plasticity, motor unit physiology, sciatic nerve action potential velocity, and motor behavior. Deficits in MUNE indicated loss of the functional connectivity between motor neurons and muscle, one of the key features of motor neuron disease [44–46]. Significantly slower sciatic nerve action potential velocity indicated the presence of axonal pathology, consistent with axonal pathology observed in the BAC CHCHD10^R15L^ mice [32]. These electrophysiological measures were mirrored in impaired rotarod performance and grip strength in CHCHD10^R15L^ and CHCHD10^S59L^ mice. As mitochondrial health and transport is crucial to sustain synaptic function at both sides of the synapse [38–40], deficits in long-term synaptic plasticity in the form of LTP in CHCHD10^R15L^ and CHCHD10^S59L^ mice indicated reduced ability to sustain synaptic efficacy, a phenotype tied to learning and memory [58]. It is notable that restoration of CHCHD10^WT^ in TDP-43 transgenic mice (TAR4) fully rescued synaptic integrity and LTP deficits induced by TDP-43, which was underpinned by significantly reduced TDP-43 pathology in TAR4;D10^WT^ mice. These transgenic models, which express CHCHD10 variants under the control of neuron-specific mouse *PrP* promoter and correctly target to the IMS of mitochondria, therefore, represent useful tools to study CHCHD10 function and dysfunction originating from neurons of the CNS. Other reported models such as the *CHCHD10*^*S55L*^ knock-in mouse model driven by the endogenous mouse *CHCHD10* promoter [33, 35] and the BAC transgenic mice driving CHCHD10^R15L^ expression with human *CHCHD10* promoter [32] exhibit largely peripheral and to a smaller extent CNS phenotypes, which exemplify different modalities to assess CHCHD10 dysfunction in vivo. In sum, this study implicates CHCHD10-regulated proteostasis and aggregation of TDP-43 as a significant factor modulating phospho-TDP-43 pathogenesis and associated disease phenotypes in FTLD-TDP and AD. Whether proteins other than TDP-43 are subject to CHCHD10-regulated proteostasis and aggregation in mitochondria is an important open question. Finally, this study indicates that a strategy to enhance endogenous CHCHD10 level or activity could be beneficial to mitigating disorders exhibiting phospho-TDP-43 pathology.

## Supplementary Information

**Additional file 1**. CHCHD10 aggregates and TDP-43 inclusions in FTLD-TDP and AD patients’ brains. (a) Information describing human brain tissue from normal, FTLD-TDP43 and AD patients used for immunohistochemistry. (b) Representative blots of pS409/410-TDP-43, and actin from RIPA-insoluble frontal cortex brain extracts. (c) Quantification of RIPA-insoluble pS409/410-TDP-43 from human FTLD-TDP and nondemented control frontal cortex (t-test, *p<0.05, n=6 FTLD-TDP, n=6 control).

**Additional file 2**. FTD/ALS-linked CHCHD10 mutations drive CHCHD10 insolubility and TDP-43 aggregation *in vivo* and in cultured cells. (a) Mitochondria isolated from 10-month-old D10^WT^ mouse brains sub-fractionated for outer membrane (OM), intermembrane space (IMS), inner membrane (IM) and matrix and then immunoblotted for hTDP-43, Flag-CHCHD10 (M2), Tom20, mitofilin, and ClpP. Known sub-mitochondrial localization of indicated proteins in parenthesis. (b) Quantification of Flag-CHCHD10 from sub-fractionated mitochondria (OM, IMS, IM, Matrix). (c) Representative frontal cortex images of 10-month-old CHCHD10WT , CHCHD10R15L and CHCHD10S59L mice immunotained for CHCHD10 (red), NeuN (green) and DAPI (blue). (d) Negative control staining of CHCHD10-WT brain without primary antibody but with secondary antibody and DAPI. (e) RIPA-soluble extracts from the cortex of 10-month-old WT, CHCHD10^WT^, CHCHD10^R15L^, and CHCHD10^S59L^ mice immunoblotted for Flag-M2 (Flag-CHCHD10) and actin. (f) Representative frontal cortex images of 10-month-old CHCHD10^S59L^ mice immunostained for Flag-CHCHD10 (green), pS409/410-TDP-43 (red), and DAPI (blue). Panels to the right show negative controls without primary antibodies but with secondary antibodies and DAPI. (g) Equal amounts of sonicated RIPA-insoluble pellets from 10-month-old CHCHD10^WT^, CHCHD10^R15L^, and CHCHD10^S59L^ mice subjected to filter trap assay for TDP-43. (h) Quantification of captured TDP-43 aggregates from figure (S2g) (1-way ANOVA, F(2, 6)=11.81, P=0.0082, posthoc Tukey, *p<0.05, **p<0.01, n=3 mice/genotype). (i-j) Tet-inducible Hela-myc-TDP-43 cells transfected with Flag-CHCHD10^WT^, Flag-CHCHD10^R15L^, or Flag-CHCHD10^S59L^ for 48h and subjected to (i) immunoblotting for Flag-CHCHD10 and actin from RIPA-soluble and RIPA-insoluble lysates and (j) filter-trap assay for CHCHD10 and TDP-43 from sonicated RIPA-insoluble pellets. (k) Quantification of captured TDP-43 aggregates from figure (S2j) (1-way ANOVA, F(2, 6)=51.65, P=0.0002, posthoc Tukey, #p<0.001, n=3/group)

**Additional file 3**. Gliosis in WT, CHCHD10^WT^, CHCHD10^R15L^, and CHCHD10^S59L^ mice. (a) Representative images of brain sections from 10-month-old WT mice immunostained for synaptophysin (red). Negative controls without primary antibody but with secondary antibody and DAPI in the right panels. (b,c) Representative images of brain sections from 10-month-old WT, CHCHD10^WT^, CHCHD10^R15L^, and CHCHD10^S59L^ mice immunostained for GFAP (green), Iba1 (red), and DAPI (blue). (d,e) Quantification of GFAP and Iba1 intensities from figure (S3b) (1-way ANOVA: (e) F(3, 96)=34.18, P<0.0001, posthoc Tukey, *p<0.05, #p<0.001, ##p<0.0001, F(3, 102)=0.5214, P=0.6685; n=22-28 sections/genotype from 4 mice/genotype)

**Additional file 4**. Increased TDP-43 pathology in the spinal cord of CHCHD10^R15L^ and CHCHD10^S59L^ mice. (a) Representative images of lumbar spinal cord sections from 10-month-old WT, CHCHD10^WT^, CHCHD10^R15L^, and CHCHD10^S59L^ mice immunostained for Flag-CHCHD10 and ChAT. (b) Representative images of lumbar spinal cord sections from 10-month-old CHCHD10^WT^, CHCHD10^R15L^, and CHCHD10^S59L^ mice immunostained for pS409/410-TDP-43 (green) and DAPI (blue). White boxes magnified in bottom panels. (c) Quantification of pS409/410-TDP-43 intensity from figure (S4b) (1-way ANOVA, F(2, 67)=11.52, ##P<0.0001: posthoc Tukey, #p<0.0005, n=23-24 images/genotype from 4 mice/genotype)

**Additional file 5**. CHCHD10^WT^ mitigates TDP-43 pathology in spinal cord of TDP-43 transgenic mice. (a) Representative images of brains section from 10-month-old TAR4;D10^WT^ mice immunostained for hTDP-43 (green), CHCHD10 (red), and DAPI (blue) in the left panel. Negative control without primary antibodies but with secondary antibodies and DAPI in the right panel. (b) Representative images of brains section from 10-month-old TAR4 mice immunostained for pS409/410-TDP-43 (green) and DAPI (blue) in the left panel. Negative control without primary antibody but with secondary antibody and DAPI in the right panel. (c) Representative images of lumbar spinal cord sections from 10-month old TAR4 and TAR4;D10^WT^ mice immunostained for hTDP-43 (green), CHCHD10 (red) and DAPI (blue). White boxes magnified in right panels. (d) Quantification of cytoplasmic to nuclear TDP-43 intensity ratio from figure (S5c) (t-test, ##p<0.0001, n=29-30 sections/genotype from 4 mice/genotype). (e) Representative images of lumbar spinal cord sections from 10-month-old TAR4 and TAR4;D10^WT^ mice immunostained for human pS409/410-TDP-43 (green) and DAPI (blue). White boxes magnified in right panels. (f) Quantification of pS409/410-TDP-43 intensity from figure (S5e) (t-test, *p<0.05, n=33-36 sections/genotype from 4 mice/genotype)

**Additional file 6**. Short-term synaptic efficacy in WT, TAR4 and TAR4;D10^WT^ mice. Acute brain slices from 10-month-old TAR4 and TAR4;D10^WT^ mice subjected to Input/Output (I/O) measurements by stepping up stimulation amplitude from 0.5 to 18mV (2-way ANOVA, genotype F(2, 3640)=49.95, P<0.0001, posthoc Tukey, *p<0.05 (WT vs. TAR4:D10^WT^: 5-5.5mV, 6.5-8.5mV, 9.5mV), not significant for other mVs among genotypes, n=24-58 slices/genotype from 3-5 mice/genotype)

**Additional file 7**. Increased mitochondrial accumulation of TDP-43 by FTD/ALS-linked mutations *in vivo* and primary neurons. (a) Isolated mitochondria from 10-month-old CHCHD10^WT^, CHCHD10^R15L^, and CHCHD10^S59L^ mice immunoblotted for CHCHD10, TDP-43, and Tom20. (b) Quantification of the mitochondrial TDP-43 from figure (S7a) (1-way ANOVA, F(2, 6)=26.43, P=0.0011: posthoc Tukey, *p<0.05 #p<0.001, n=3 mice/genotype). (c) Representative images of cortical primary neurons derived from WT, CHCHD10^WT^, CHCHD10^R15L^, and CHCHD10^S59L^ mice transduced with TDP-43-tomato-HA AAV9 on DIV7, immunostained for human Tom20 (green), and subjected to direct immunofluorescence for TDP-43-tomato (red) on DIV21. White boxes magnified in bottom panels. (d) Quantification of cytoplasmic TDP-43-tomato colocalized with Tom20 from figure (S7c) (1-way ANOVA, F(3, 31)=27.1, P<0.0001: posthoc Tukey, *p<0.05, #p<0.001, ##p<0.0001, n=5-15 images/genotype)

**Additional file 8**. Purification of recombinant CHCHD10^WT^ and CHCHD10^S59L^. Representative Coomassie blue stain of CHCHD10^WT^ and CHCHD10^S59L^ proteins purified from *E. Coli*.

## Data Availability

Data supporting the findings of this study are available from the corresponding authors on reasonable request.
