# Supplementary figures and images for: Modulation of synaptic plasticity, motor unit physiology, and TDP-43 pathology by CHCHD10
Source: Acta Neuropathol Commun. 2022 Jul 4;10:95. doi: 10.1186/s40478-022-01386-9 (PMC9254494; doi:10.1186/s40478-022-01386-9)

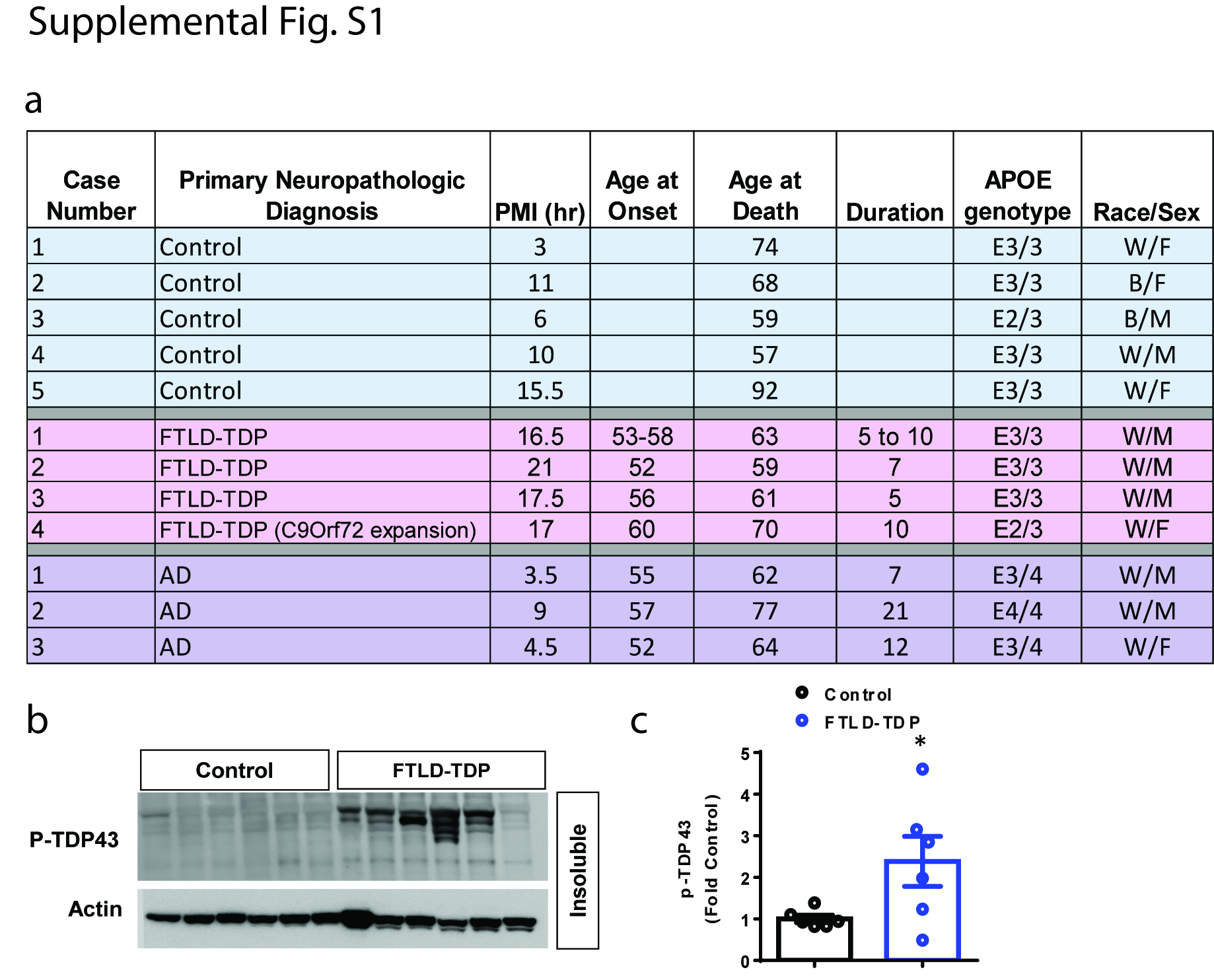

Supplement: Supplementary file 1 — Additional file 1. CHCHD10 aggregates and TDP-43 inclusions in FTLD-TDP and AD patients’ brains. (a) Information describing human brain tissue from normal, FTLD-TDP43 and AD patients used for immunohistochemistry. (b) Representative blots of pS409/410-TDP-43, and actin from RIPA-insoluble frontal cortex brain extracts. (c) Quantification of RIPA-insoluble pS409/410-TDP-43 from human FTLD-TDP and nondemented control frontal cortex (t-test, *p<0.05, n=6 FTLD-TDP, n=6 control). [file 40478_2022_1386_MOESM1_ESM.tif]

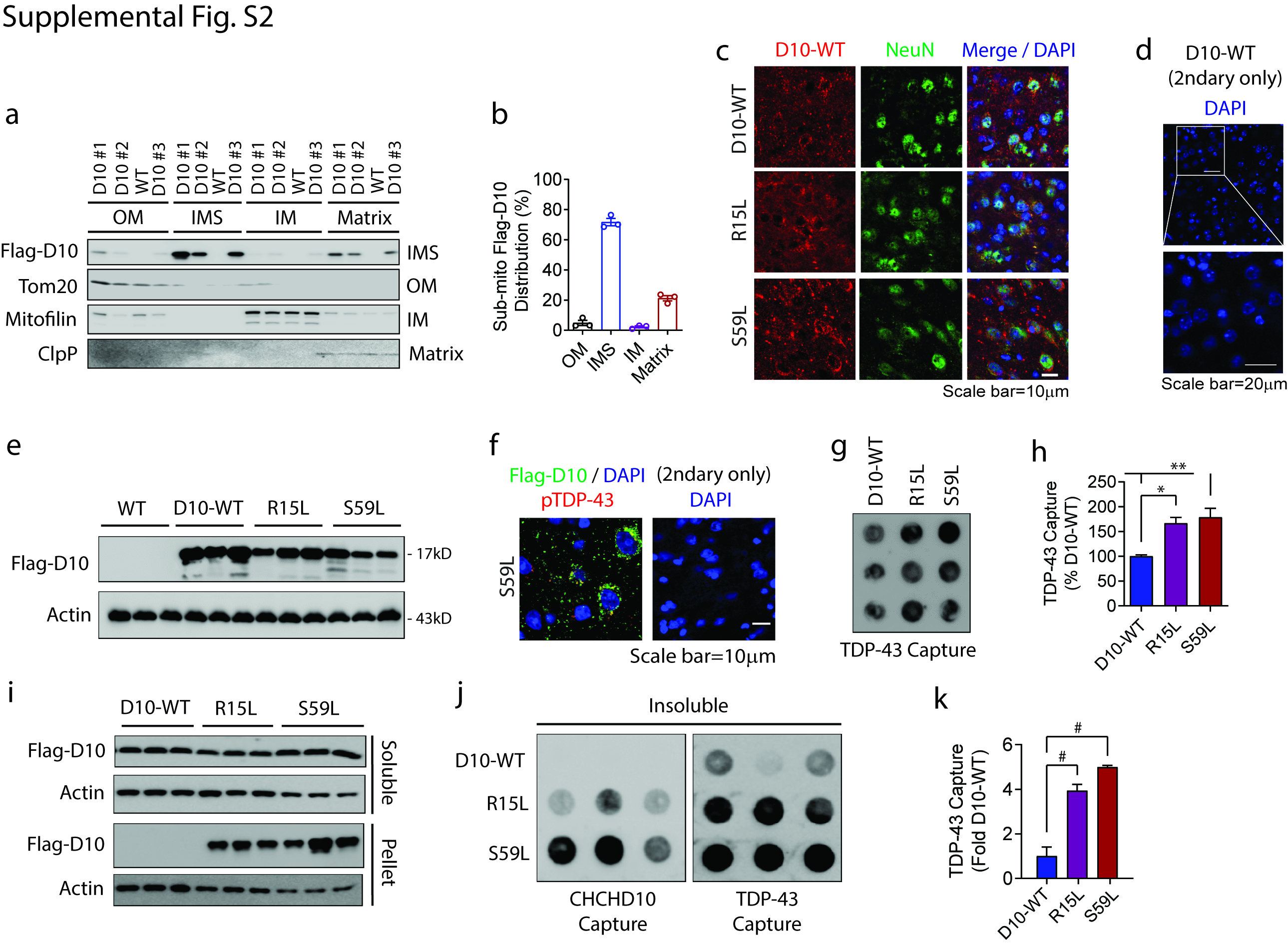

Supplement: Supplementary file 2 — Additional file 2. FTD/ALS-linked CHCHD10 mutations drive CHCHD10 insolubility and TDP-43 aggregation in vivo and in cultured cells. (a) Mitochondria isolated from 10-month-old D10WT mouse brains sub-fractionated for outer membrane (OM), intermembrane space (IMS), inner membrane (IM) and matrix and then immunoblotted for hTDP-43, Flag-CHCHD10 (M2), Tom20, mitofilin, and ClpP. Known sub-mitochondrial localization of indicated proteins in parenthesis. (b) Quantification of Flag-CHCHD10 from sub-fractionated mitochondria (OM, IMS, IM, Matrix). (c) Representative frontal cortex images of 10-month-old CHCHD10WT , CHCHD10R15L and CHCHD10S59L mice immunotained for CHCHD10 (red), NeuN (green) and DAPI (blue). (d) Negative control staining of CHCHD10-WT brain without primary antibody but with secondary antibody and DAPI. (e) RIPA-soluble extracts from the cortex of 10-month-old WT, CHCHD10WT, CHCHD10R15L, and CHCHD10S59L mice immunoblotted for Flag-M2 (Flag-CHCHD10) and actin. (f) Representative frontal cortex images of 10-month-old CHCHD10S59L mice immunostained for Flag-CHCHD10 (green), pS409/410-TDP-43 (red), and DAPI (blue). Panels to the right show negative controls without primary antibodies but with secondary antibodies and DAPI. (g) Equal amounts of sonicated RIPA-insoluble pellets from 10-month-old CHCHD10WT, CHCHD10R15L, and CHCHD10S59L mice subjected to filter trap assay for TDP-43. (h) Quantification of captured TDP-43 aggregates from figure (S2g) (1-way ANOVA, F(2, 6)=11.81, P=0.0082, posthoc Tukey, *p<0.05, **p<0.01, n=3 mice/genotype). (i-j) Tet-inducible Hela-myc-TDP-43 cells transfected with Flag-CHCHD10WT, Flag-CHCHD10R15L, or Flag-CHCHD10S59L for 48h and subjected to (i) immunoblotting for Flag-CHCHD10 and actin from RIPA-soluble and RIPA-insoluble lysates and (j) filter-trap assay for CHCHD10 and TDP-43 from sonicated RIPA-insoluble pellets. (k) Quantification of captured TDP-43 aggregates from figure (S2j) (1-way ANOVA, F(2, 6)=51.65, P=0.0002, posthoc T [file 40478_2022_1386_MOESM2_ESM.tif]

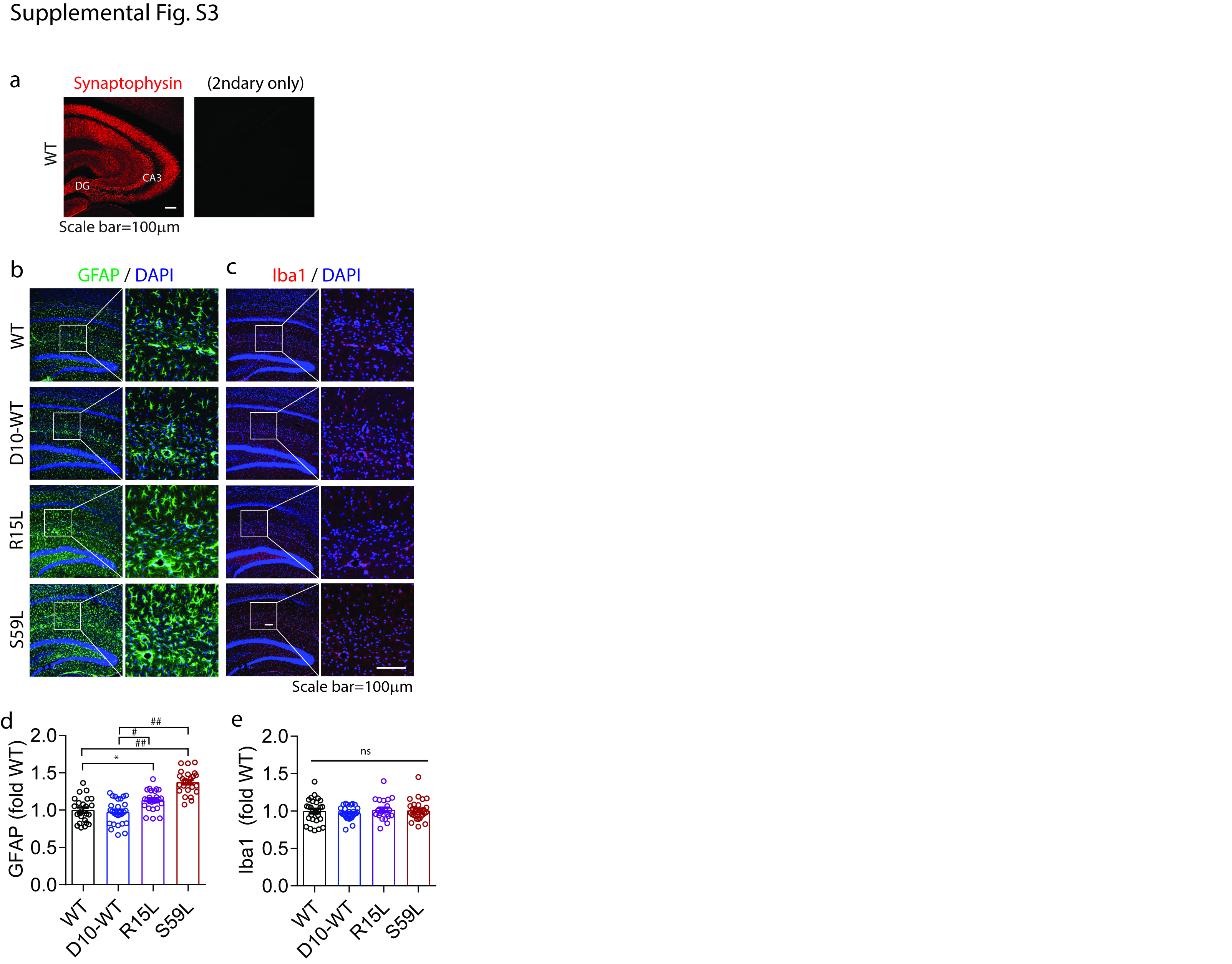

Supplement: Supplementary file 3 — Additional file 3. Gliosis in WT, CHCHD10WT, CHCHD10R15L, and CHCHD10S59L mice. (a) Representative images of brain sections from 10-month-old WT mice immunostained for synaptophysin (red). Negative controls without primary antibody but with secondary antibody and DAPI in the right panels. (b,c) Representative images of brain sections from 10-month-old WT, CHCHD10WT, CHCHD10R15L, and CHCHD10S59L mice immunostained for GFAP (green), Iba1 (red), and DAPI (blue). (d,e) Quantification of GFAP and Iba1 intensities from figure (S3b) (1-way ANOVA: (e) F(3, 96)=34.18, P<0.0001, posthoc Tukey, *p<0.05, #p<0.001, ##p<0.0001, F(3, 102)=0.5214, P=0.6685; n=22-28 sections/genotype from 4 mice/genotype) [file 40478_2022_1386_MOESM3_ESM.tif]

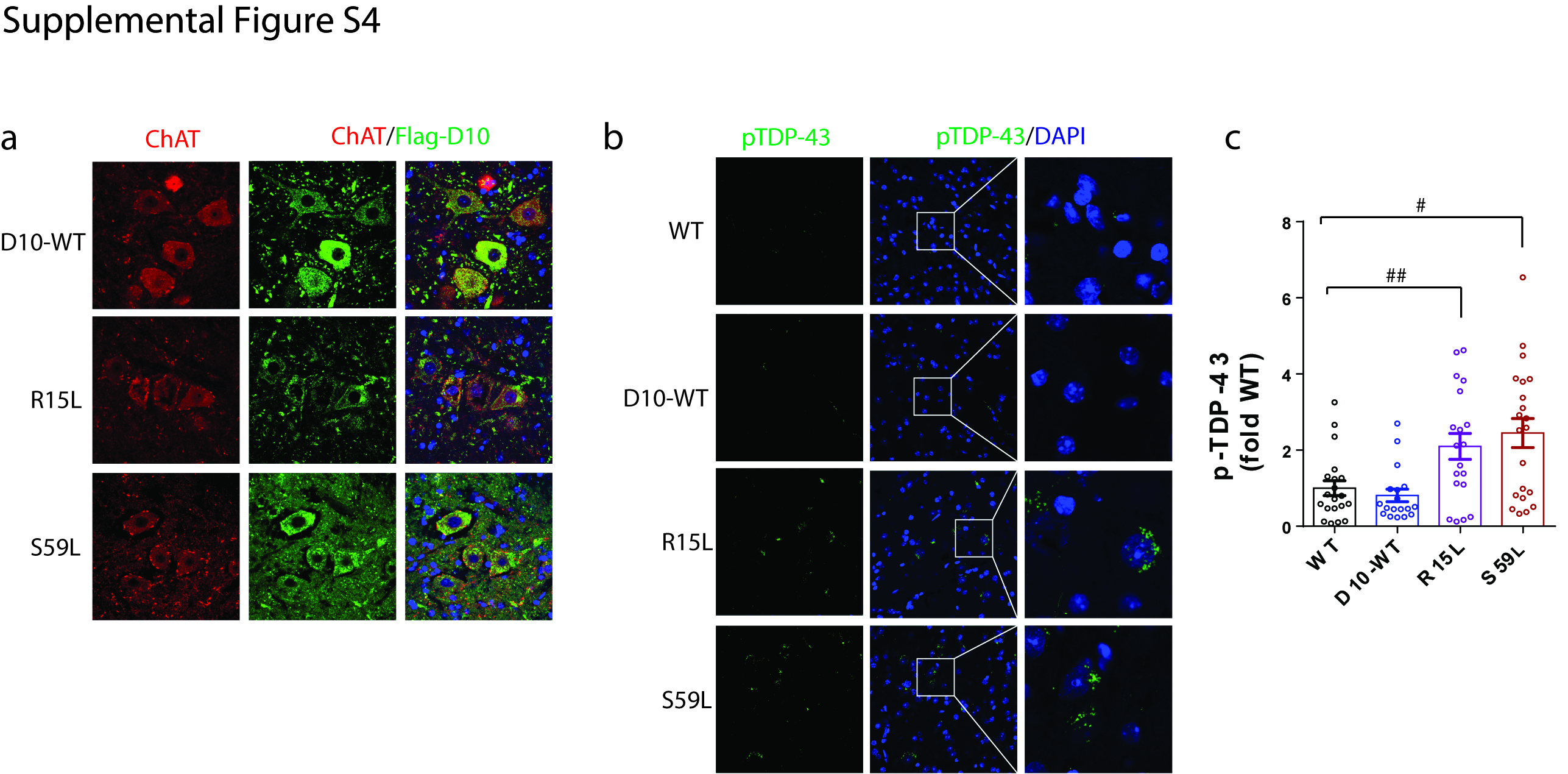

Supplement: Supplementary file 4 — Additional file 4. Increased TDP-43 pathology in the spinal cord of CHCHD10R15L and CHCHD10S59L mice. (a) Representative images of lumbar spinal cord sections from 10-month-old WT, CHCHD10WT, CHCHD10R15L, and CHCHD10S59L mice immunostained for Flag-CHCHD10 and ChAT. (b) Representative images of lumbar spinal cord sections from 10-month-old CHCHD10WT, CHCHD10R15L, and CHCHD10S59L mice immunostained for pS409/410-TDP-43 (green) and DAPI (blue). White boxes magnified in bottom panels. (c) Quantification of pS409/410-TDP-43 intensity from figure (S4b) (1-way ANOVA, F(2, 67)=11.52, ##P<0.0001: posthoc Tukey, #p<0.0005, n=23-24 images/genotype from 4 mice/genotype) [file 40478_2022_1386_MOESM4_ESM.tif]

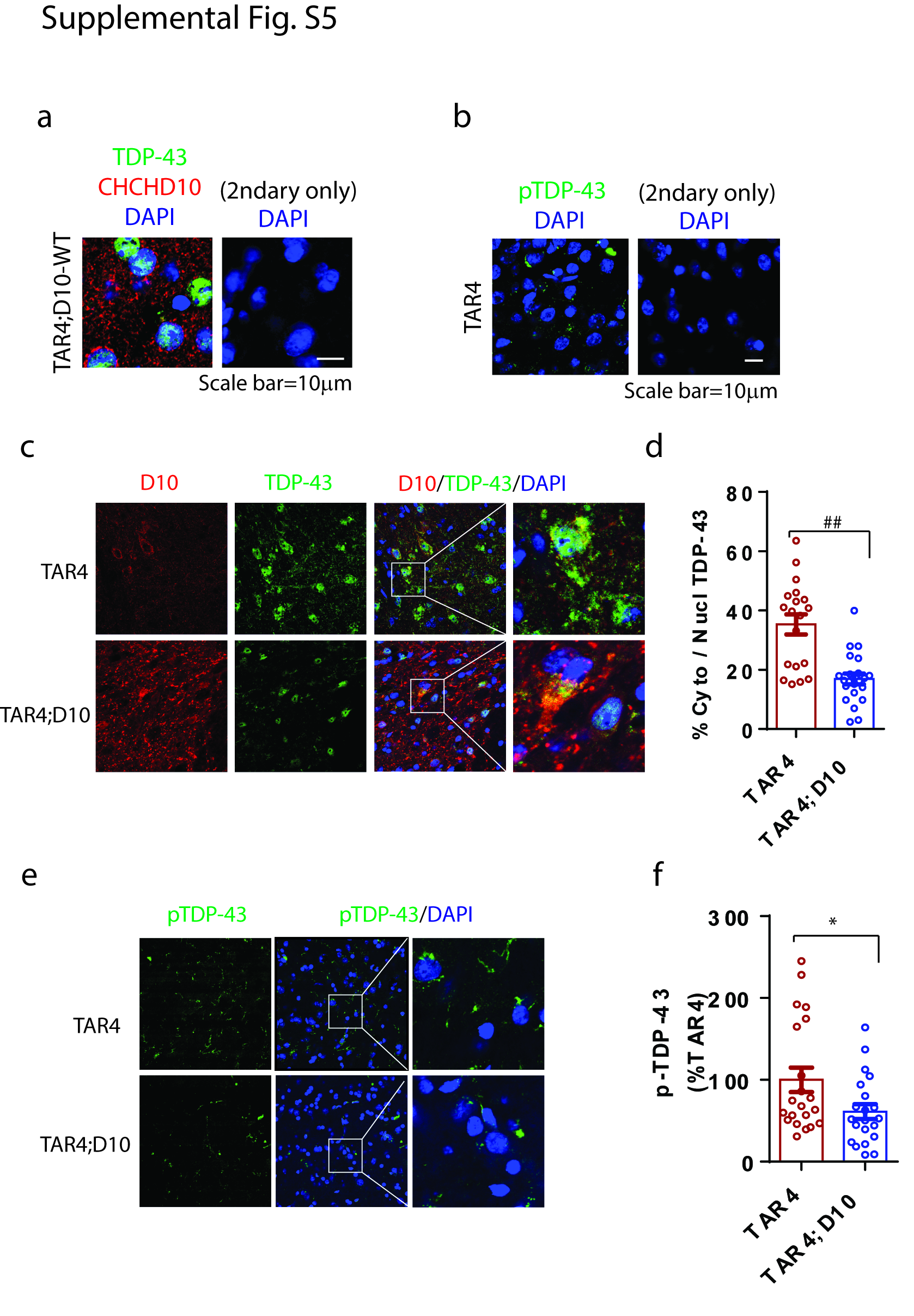

Supplement: Supplementary file 5 — Additional file 5. CHCHD10WT mitigates TDP-43 pathology in spinal cord of TDP-43 transgenic mice. (a) Representative images of brains section from 10-month-old TAR4;D10WT mice immunostained for hTDP-43 (green), CHCHD10 (red), and DAPI (blue) in the left panel. Negative control without primary antibodies but with secondary antibodies and DAPI in the right panel. (b) Representative images of brains section from 10-month-old TAR4 mice immunostained for pS409/410-TDP-43 (green) and DAPI (blue) in the left panel. Negative control without primary antibody but with secondary antibody and DAPI in the right panel. (c) Representative images of lumbar spinal cord sections from 10-month old TAR4 and TAR4;D10WT mice immunostained for hTDP-43 (green), CHCHD10 (red) and DAPI (blue). White boxes magnified in right panels. (d) Quantification of cytoplasmic to nuclear TDP-43 intensity ratio from figure (S5c) (t-test, ##p<0.0001, n=29-30 sections/genotype from 4 mice/genotype). (e) Representative images of lumbar spinal cord sections from 10-month-old TAR4 and TAR4;D10WT mice immunostained for human pS409/410-TDP-43 (green) and DAPI (blue). White boxes magnified in right panels. (f) Quantification of pS409/410-TDP-43 intensity from figure (S5e) (t-test, *p<0.05, n=33-36 sections/genotype from 4 mice/genotype) [file 40478_2022_1386_MOESM5_ESM.tif]

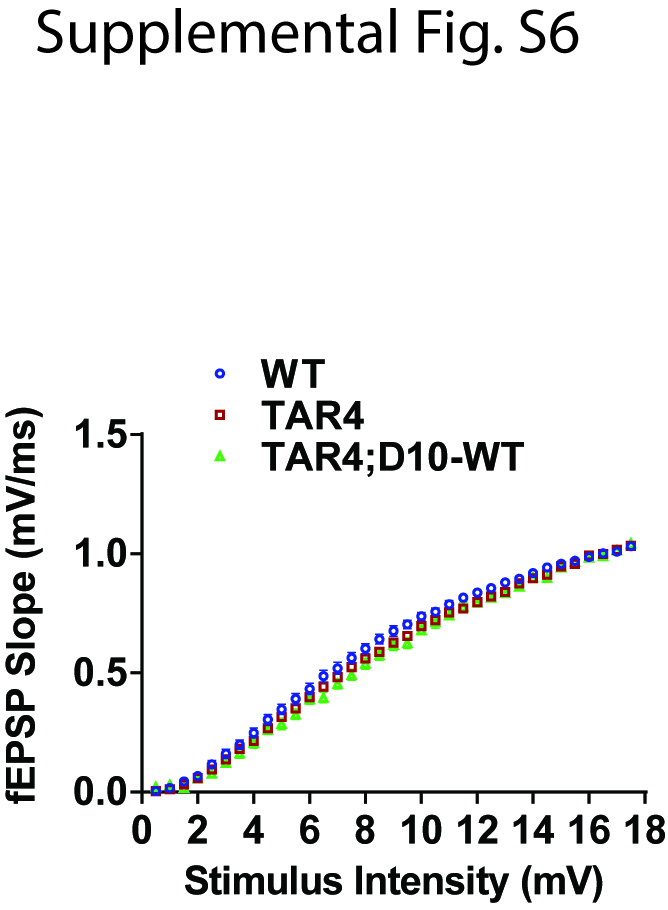

Supplement: Supplementary file 6 — Additional file 6. Short-term synaptic efficacy in WT, TAR4 and TAR4;D10WT mice. Acute brain slices from 10-month-old TAR4 and TAR4;D10WT mice subjected to Input/Output (I/O) measurements by stepping up stimulation amplitude from 0.5 to 18mV (2-way ANOVA, genotype F(2, 3640)=49.95, P<0.0001, posthoc Tukey, *p<0.05 (WT vs. TAR4:D10WT: 5-5.5mV, 6.5-8.5mV, 9.5mV), not significant for other mVs among genotypes, n=24-58 slices/genotype from 3-5 mice/genotype) [file 40478_2022_1386_MOESM6_ESM.tif]

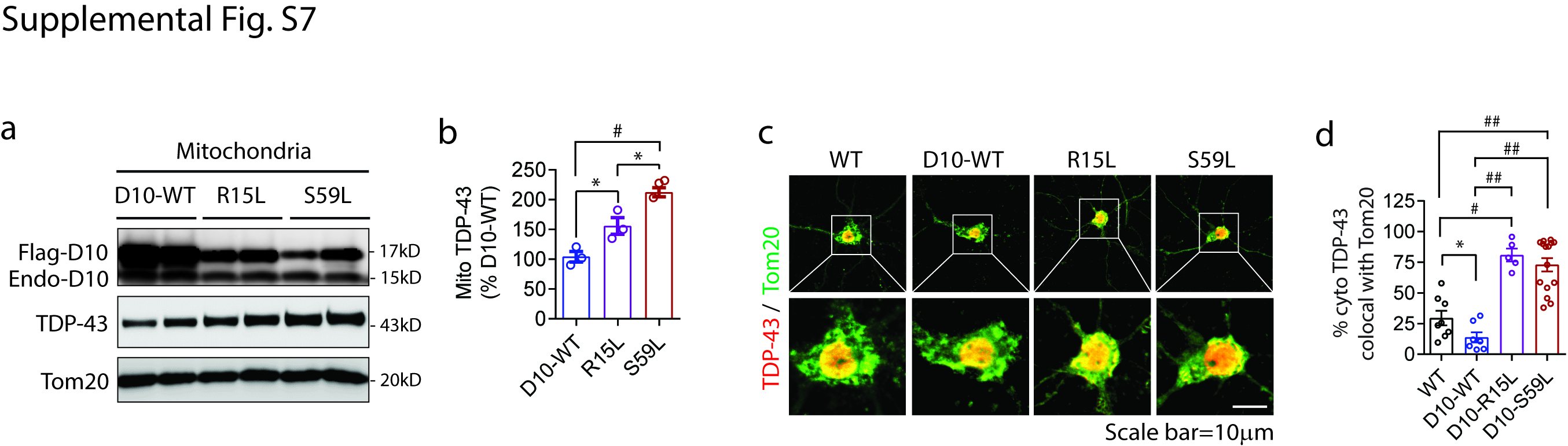

Supplement: Supplementary file 7 — Additional file 7. Increased mitochondrial accumulation of TDP-43 by FTD/ALS-linked mutations in vivo and primary neurons. (a) Isolated mitochondria from 10-month-old CHCHD10WT, CHCHD10R15L, and CHCHD10S59L mice immunoblotted for CHCHD10, TDP-43, and Tom20. (b) Quantification of the mitochondrial TDP-43 from figure (S7a) (1-way ANOVA, F(2, 6)=26.43, P=0.0011: posthoc Tukey, *p<0.05 #p<0.001, n=3 mice/genotype). (c) Representative images of cortical primary neurons derived from WT, CHCHD10WT, CHCHD10R15L, and CHCHD10S59L mice transduced with TDP-43-tomato-HA AAV9 on DIV7, immunostained for human Tom20 (green), and subjected to direct immunofluorescence for TDP-43-tomato (red) on DIV21. White boxes magnified in bottom panels. (d) Quantification of cytoplasmic TDP-43-tomato colocalized with Tom20 from figure (S7c) (1-way ANOVA, F(3, 31)=27.1, P<0.0001: posthoc Tukey, *p<0.05, #p<0.001, ##p<0.0001, n=5-15 images/genotype) [file 40478_2022_1386_MOESM7_ESM.tif]

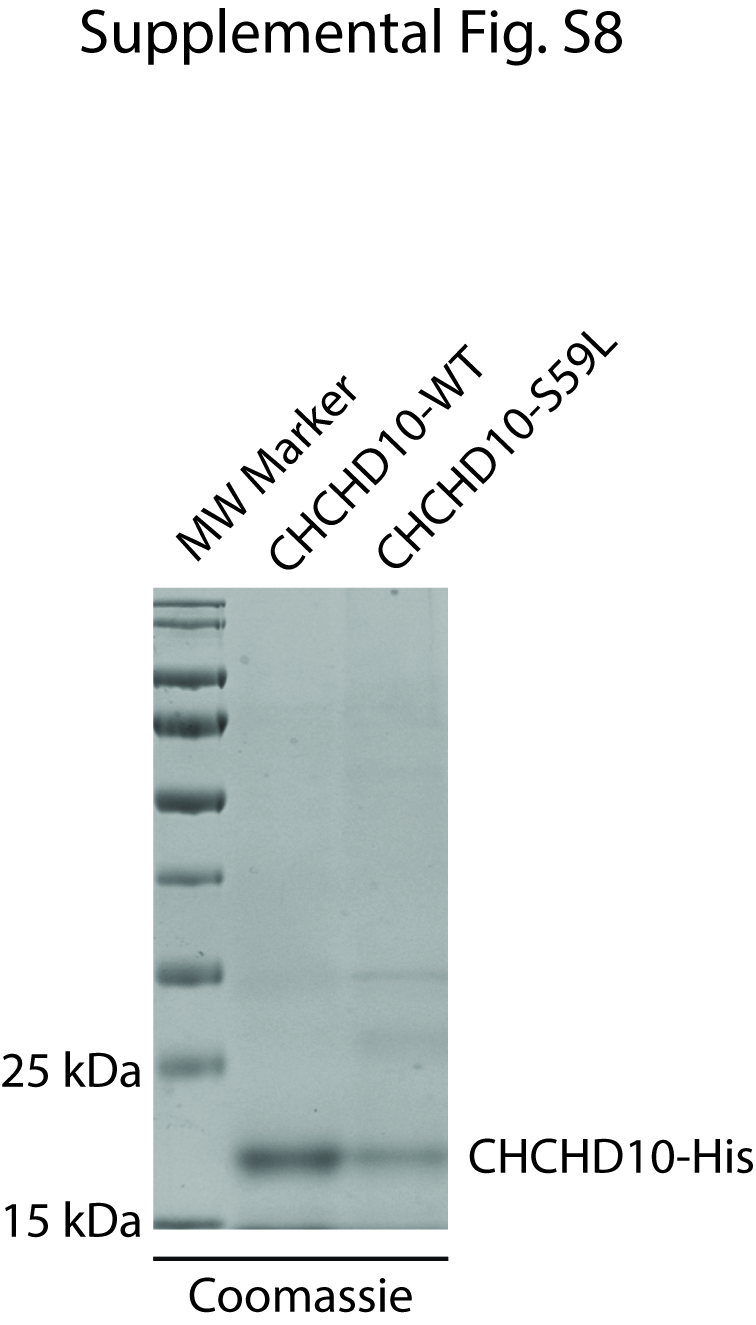

Supplement: Supplementary file 8 — Additional file 8. Purification of recombinant CHCHD10WT and CHCHD10S59L. Representative Coomassie blue stain of CHCHD10WT and CHCHD10S59L proteins purified from E. Coli. [file 40478_2022_1386_MOESM8_ESM.tif]
